# Supplementary material for: Dissecting aneuploidy phenotypes by constructing Sc2.0 chromosome VII and SCRaMbLEing synthetic disomic yeast
Source: Cell Genom. 2023 Nov 9;3(11):100364. doi: 10.1016/j.xgen.2023.100364 (PMC10667312; doi:10.1016/j.xgen.2023.100364)
Supplement: Document S1. Figures S1–S11 and Tables S1 and S2 [file mmc1.pdf]

Cell Genomics, Volume *3*

Supplemental information

Dissecting aneuploidy phenotypes by constructing Sc2.0 chromosome VII and SCRaMbLEing synthetic disomic yeast

Yue Shen, Feng Gao, Yun Wang, Yuerong Wang, Ju Zheng, Jianhui Gong, Jintao Zhang, Zhouqing Luo, Daniel Schindler, Yang Deng, Weichao Ding, Tao Lin, Reem Swidah, Hongcui Zhao, Shuangying Jiang, Cheng Zeng, Shihong Chen, Tai Chen, Yong Wang, Yisha Luo, Leslie Mitchell, Joel S. Bader, Guojie Zhang, Xia Shen, Jian Wang, Xian Fu, Junbiao Dai, Jef D. Boeke, Huanming Yang, Xun Xu, and Yizhi Cai

Supplemental information

Dissecting aneuploidy phenotypes by constructing Sc2.0 chromosome VII and SCRaMbLEing synthetic disomic yeast

Yue Shen, Feng Gao, Yun Wang, Yuerong Wang, Ju Zheng, Jianhui Gong, Jintao Zhang, Zhouqing Luo, Daniel Schindler, Yang Deng, Weichao Ding, Tao Lin, Reem Swidah, Hongcui Zhao, Shuangying Jiang, Cheng Zeng, Shihong Chen, Tai Chen, Yong Wang, Yisha Luo, Leslie Mitchell, Joel S. Bader, GuojieZhang, Xia Shen, Jian Wang, Xian Fu, Junbiao Dai, Jef D. Boeke, Huanming Yang, Xun Xu, and Yizhi Cai

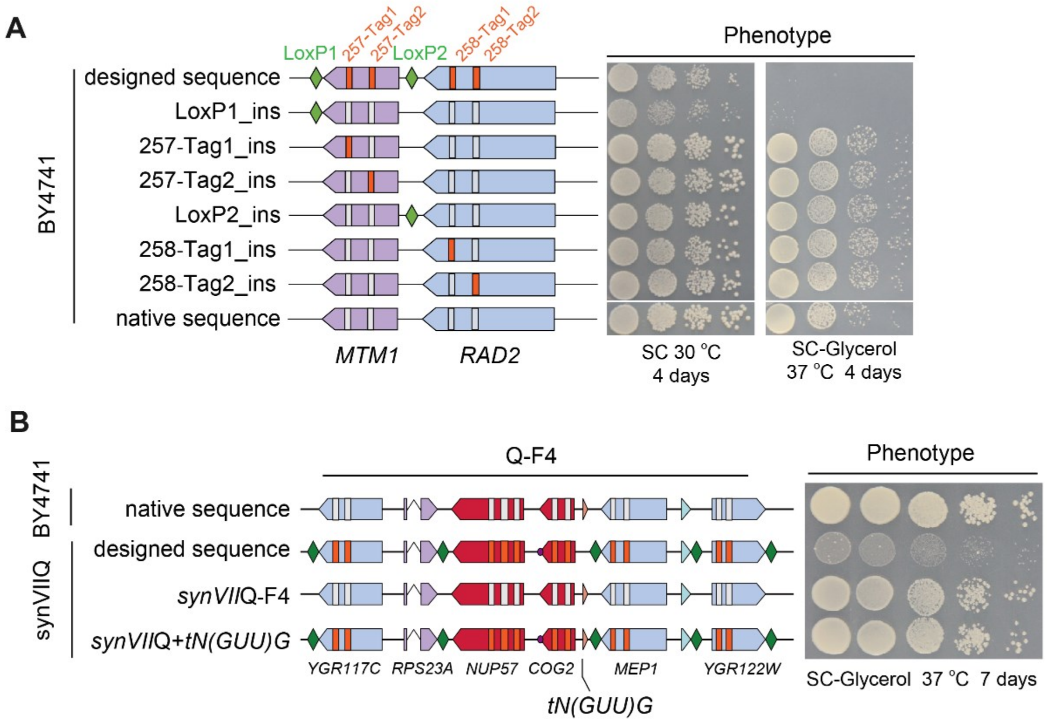

Figure S1. Debugging of W and Q regions in *synVII*, related to Figure 1. (A) Systematic dissection of defect origin in megachunk W by introducing each individual design feature into the BY4741 strain for phenotypic assay showing the loxPsym site at the 3'-UTR of *MTM1* causes the defect. (B) Spotting assay with ten-fold serial dilutions of BY4741, synVIIQ intermediate strain andits derived strains with chunk 4 and tRNA gene *tN(GUU)G* in chunk 4 replaced by native sequence showing the addition of tRNA gene *tN(GUU)G* recovered the phenotype. The arrows represent gene order and orientations (red indicates essential genes, purple is fast-growth and blue represents non-essential). Green diamonds represent loxPsym sites embedded downstream of the stop codons. Vertical orange and white bars represent synthetic and wild-type PCRTags respectively.

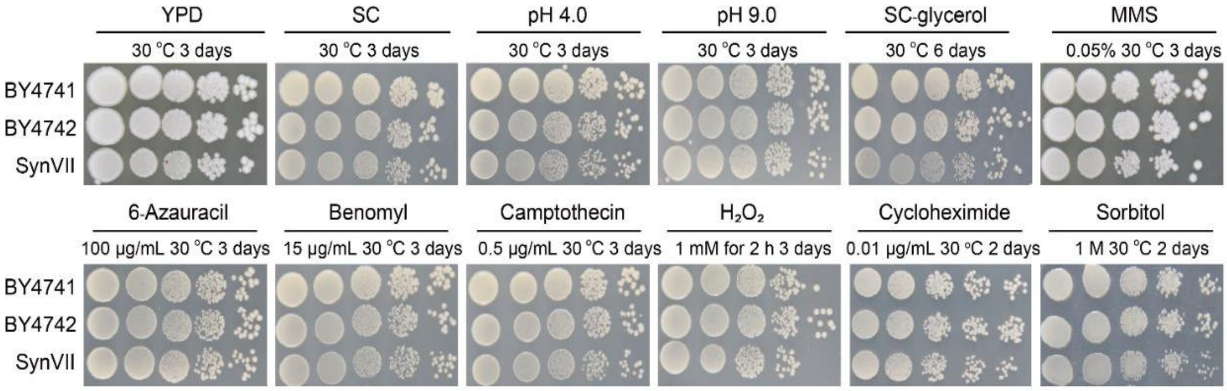

Figure S2. Phenotypic assays of synVII on different media in comparison with wild-type BY4741 and BY4742, related to Figure 1F and 1G. 10-fold serial dilutions of overnight cultures of selected strains were used for plating. From left to right: YPD at 30 °C; SC at 30 °C; low pH YPD (pH 4.0); high pH YPD (pH 9.0); SC-Glycerol; YPD + MMS; SC + 6-azauracil; SC + benomyl;SC + camptothecin; SC + H_2O_2 (1 mM, 2 hours pretreatment); SC + cycloheximide; SC + sorbitol; (YPD, yeast extract peptone dextrose; SC, synthetic complete; MMS, methyl methanesulfonate).

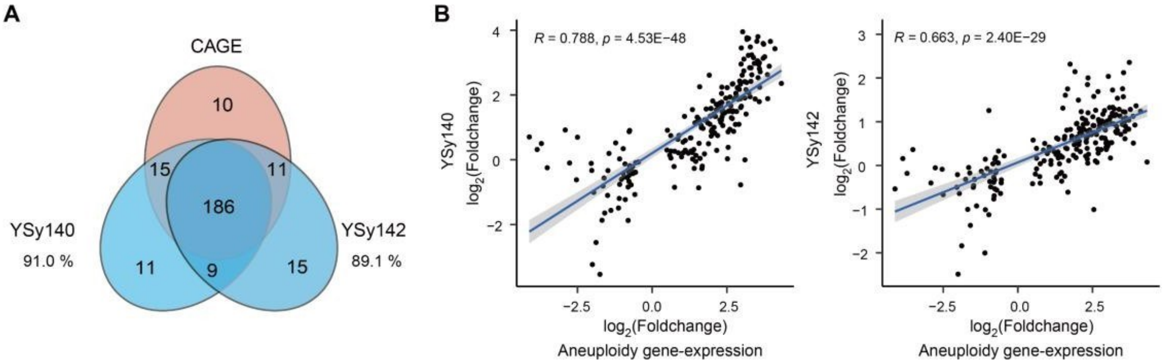

Figure S3. Comparison of the aneuploidy gene-expression in CAGE, related to Figure 2C. (A) Venn diagrams showing common genes with consistent differential gene expression among YSy140, YSy142 and aneuploidy gene-expression in CAGE. The percentage represents the consistency rate. The differential gene expression of the aneuploidy gene-expression, CAGE genes (n=222) originate from a previous study[10]. CAGE, common aneuploidy gene-expression. (**B**) Correlation of gene differential expression in YSy140 and YSy142 compared with aneuploidy gene-expression in CAGE genes. R indicates the Pearson's correlation. Solid blue line: fitted curve (ggplot:geom_point), geom_smooth (method=lm, se=TRUE). Gray area: 95% confidence range for fitted curve.

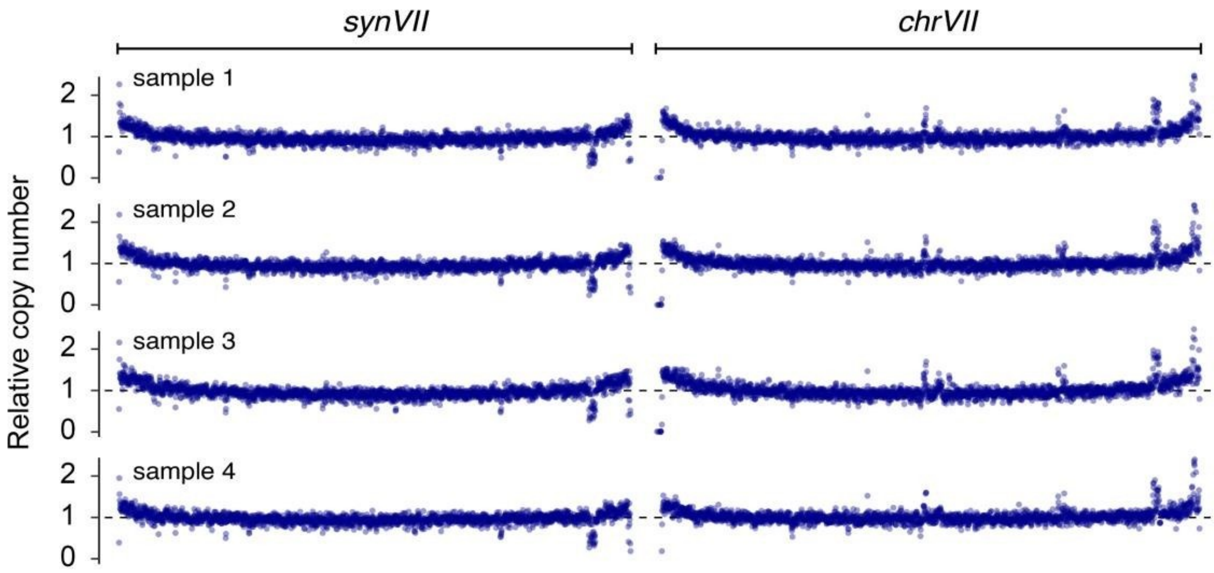

Figure S4. Verification of sequence integrity in disomic strain YSy142, related to Figure 2D. The relative sequencing depth of YSy142 colonies after batch transfer for 220 generations. The relative sequencing depth was calculated in 500-bp windows by comparing to the average of sequencing depth of the whole genome.

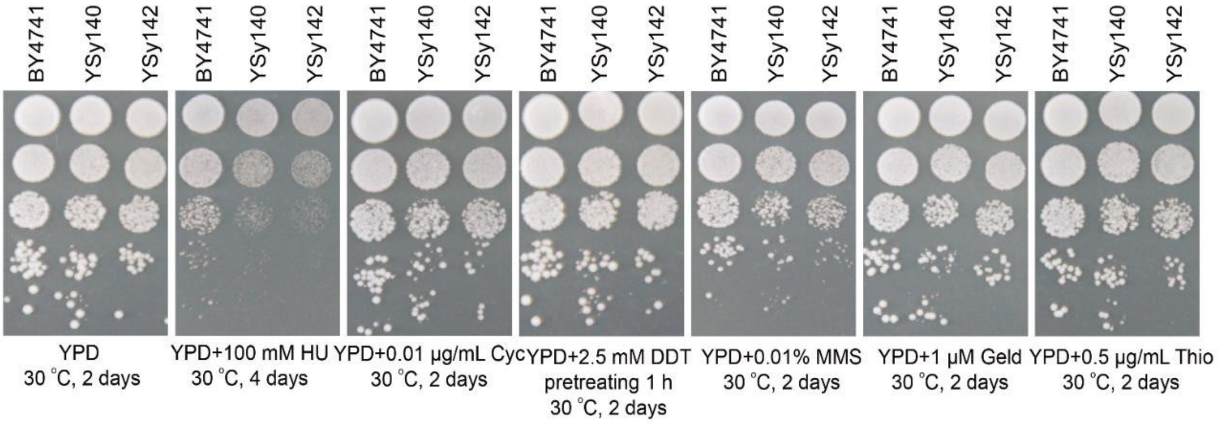

Figure S5. Spotting assay with ten-fold serial dilutions of disomic yeasts YSy140 and YSy142 compared to euploid strain BY4741 under selected conditions, related to Figure 3A. From leftto right: YPD at 30 °C; YPD + hydroxyurea (HU, 100 mM) at 30 °C; YPD + cycloheximide (Cyc, 0.01 μg/mL) at 30 °C; YPD + DL-Dithiothreitol (DDT, 2.5 mM pretreating 1 h); YPD + methyl methanesulfonate (MMS, 0.01% v/v) at 30 °C; YPD + geldanamycin (Geld, 1 μM) at 30 °C; YPD + thiolutin (Thio, 0.5 μg/mL) at 30 °C; (YPD, yeast extract peptone dextrose).

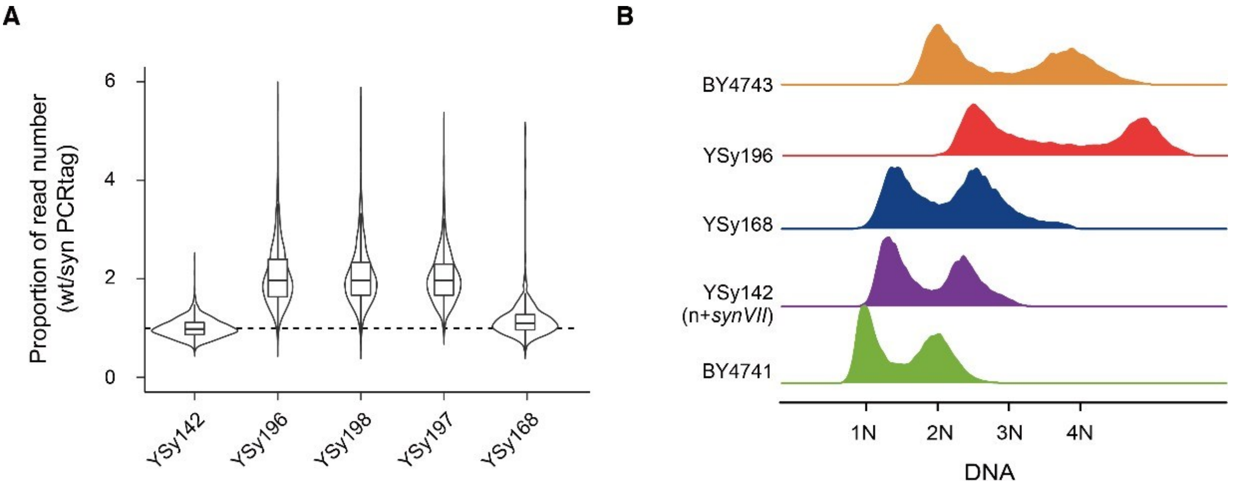

Figure S6. Karyotype analysis of SCRaMbLEd yeasts by whole genome sequencing analysis and flow cytometry, related to Figure 3. (A) The boxplot and violin plot present the proportion of reads for each wild-type (wt) versus synthetic (syn) PCRTags. The proportion is normalized by the mean of YSy142. The PCRtag sites of deleted region by SCRaMbLE is not included in the analysis. (B) For flow cytometry, asynchronous log-phase cells were analyzed (~15000 to 18000 cells/ each sample).

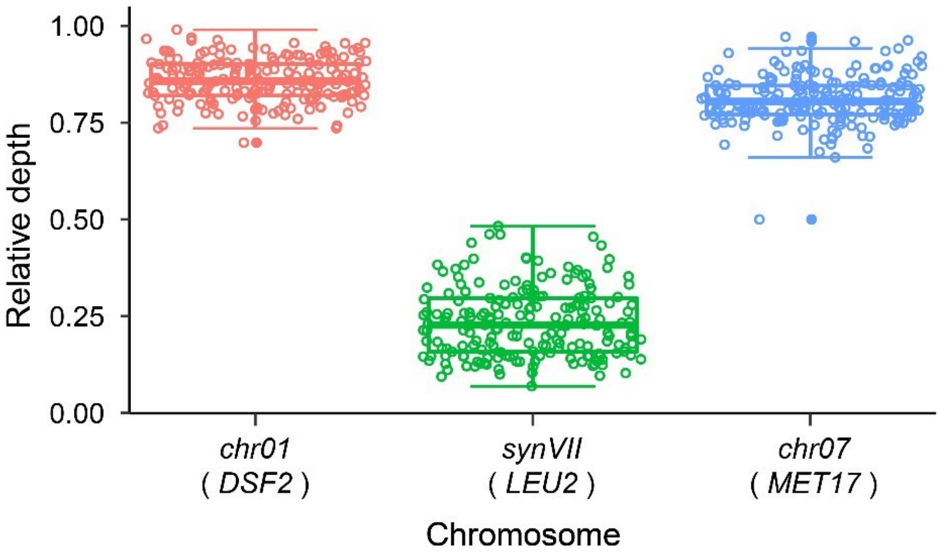

Figure S7. Relative average sequencing depth of 195 SCRaMbLEd strains with circular *synVII*, related to Figure 3. Deep sequencing coverage analysis revealed the lower depth of circular*synVII* in comparison with *chrVII* and *chrI* (control). The relative depths were quantified by *DSF2* gene on *chrI*, *LEU2* gene on *synVII* and *MET17* geneon *chrVII* respectively. Each dot represents one SCRaMbLEd strain.

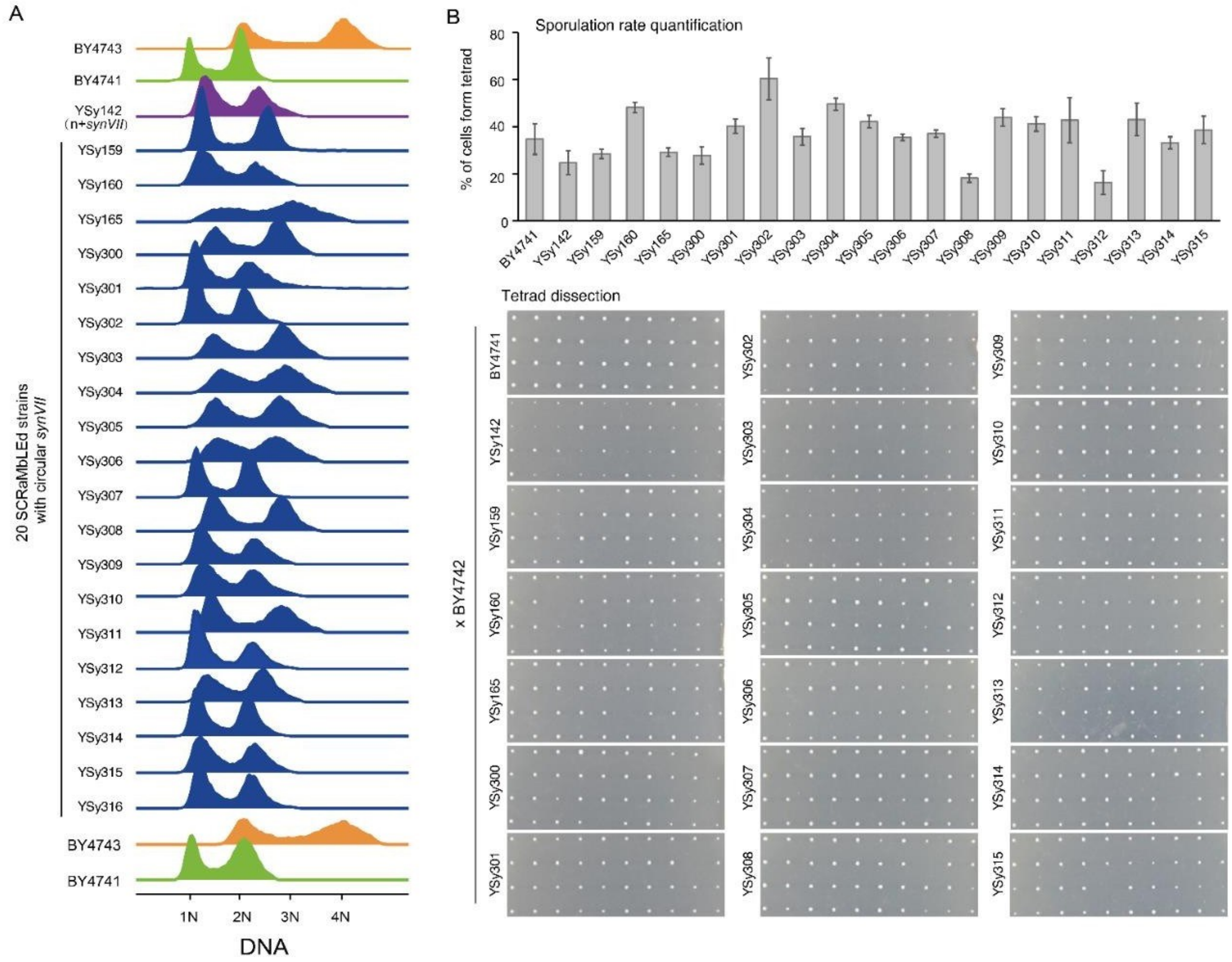

Figure S8. Karyotype validation by flow cytometry and sporulation in SCRaMbLEd strains with circular *synVII* chromosome, related to Figure 3. (A) For flow cytometry, ~15000 to 18000 cells were sorted. (B) For sporulation all selected samples were first mated to BY4742, then followed by sporulation and tetrad dissection (for each sample, 300 cells were counted at least for sporulation rate quantification, 10 tetrads were selected for dissection).

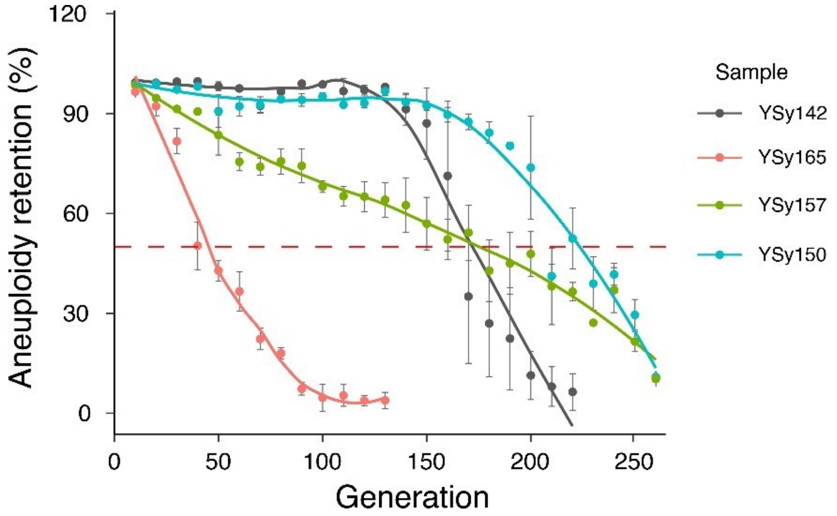

Figure S9. Genome stability analysis of SCRaMbLEd strains with circular *synVII* chromosome, related to Figure 3. Three representative strains with distinct *synVII* chromosome content and size were selected for genome stability assay by batch transfer for ~230 generations. YSy142 (n + *synVII*) is used as control.

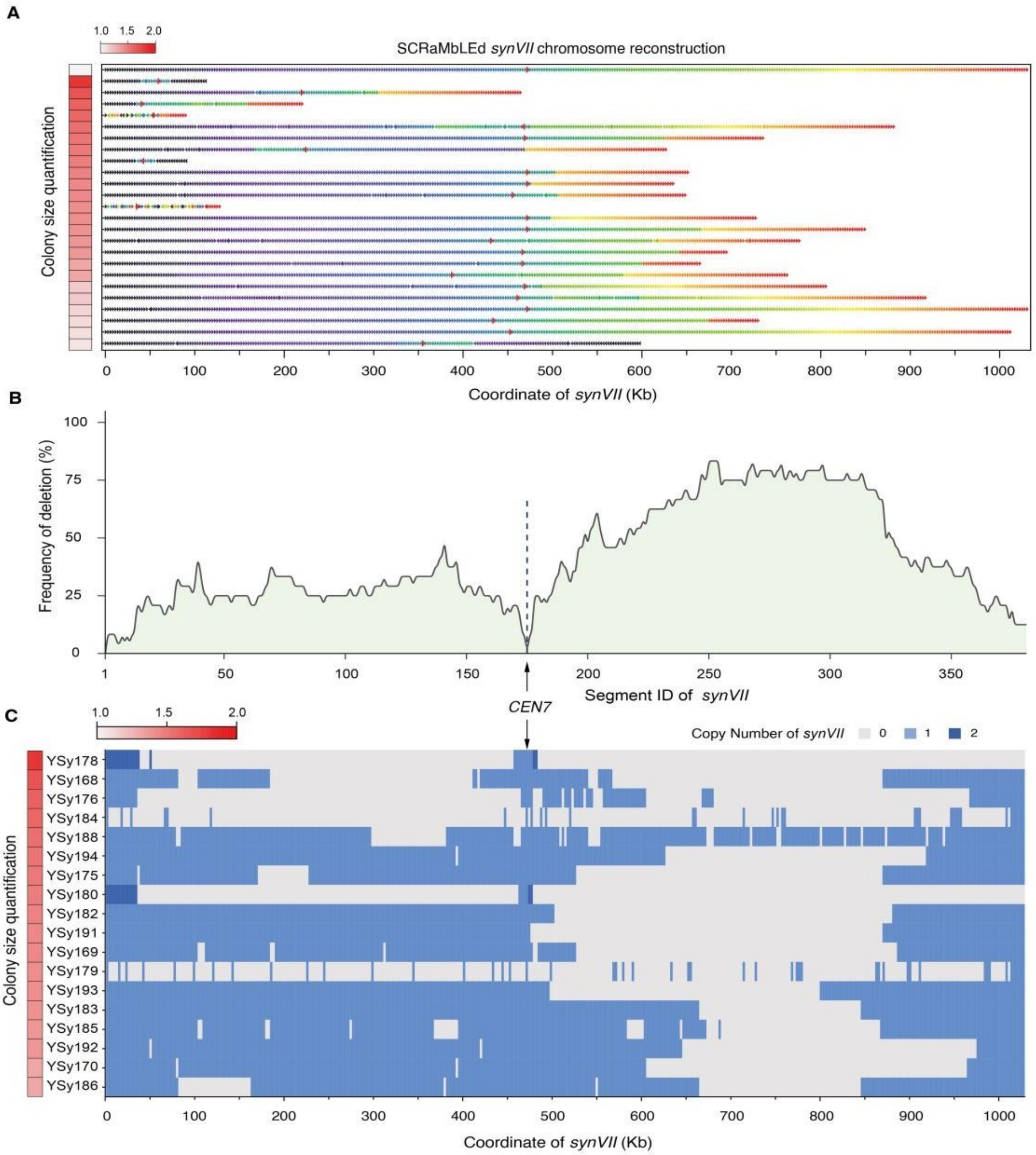

Figure S10. Deletion distribution across entire synthetic chromosome VII, related to Figure 3 and 5B. (**A**) Rearrangements were observed in the 24 SCRaMbLEd aneuploid yeasts with linear *synVII*. Each SCRaMbLE strain is represented as a sequence of arrows (SCRaMbLE-gram). The color and direction of each arrow indicates the segment number in the parental chromosome and its orientation. A red border denotes a segment containing *CEN7*. Y-axis shows the relative average phenotypic recovery rate of each SCRaMbLEd strain in comparison to that of YSy142 represented by color scale (n ≥ 200). (**B**) Deletion hotspot observed in the right arm region of *synVII*. Y-axis represents the percentage of SCRaMbLEd strains that with corresponding segment been deleted. (**C**) The fate of each segment flanked by two loxPsym sites in each strain is indicated as deleted (gray) and one copy with no change (light blue).

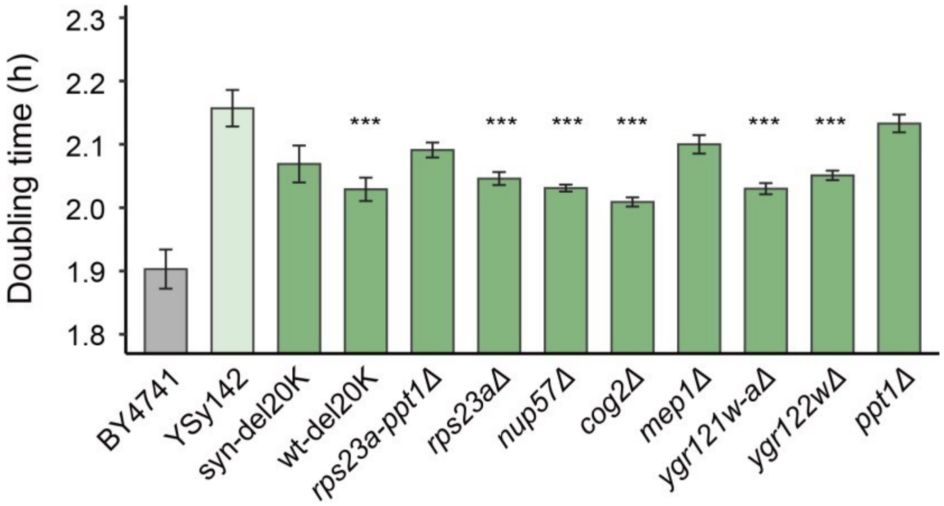

Figure S11. Doubling time measurement of reconstructed strains carrying the removal of regions/genes under normal condition, related to Figure 5C. Doubling time measurement of YSy142, *syn-del20K*, *wt-del20K*, and YSy142 carrying *syn-del12K* as well as the single gene knock-out in YPD medium at 30 °C. The error bar indicates standard error of the mean (SEM) in seven biological replicates. The P-value was calculated using single-tailed student t-test. *** P-value < 0.01.

Table S1. Summary of *synVII* design, related to STAR Methods.

Modification	Design feature	Number	Base alteration
Replacement	wild type telomere -> Universal Telomere Cap[a]	2	21094 -> 1378
	705 pairs of WT PCR tags and 705 pairs of SYN PCRTags	1410	17876
	stop codon TAG -> TAA	126	126
	"landmark" restriction sites (removal or introduction)[b]	261	596
	repeatsmash of gene	3	2147
Deletion	transposable element region[c]	20	51111
	gene	14	12232
	tRNA	36	2887
	intron[d]	23	3062
Insertion	LoxPsym site	380	12920

a. Wild-type telomeres together with its adjacent subtelomeric regions are replaced with universal telomere caps.

b. Unique restriction sites were generated in synVII either by introduction of synonymous mutations in ORFs, or by removal of redundant sites by the same method to leave only one pre- existing instance of a given site in the synVII sequence.

c. Transposable element region include these functional features of LTR retrotransposons, transposable element genes and long terminal repeats.

d. Eleven introns were retained as these introns reside in ribosomal subunit coding genes.

Table S2. Chromosome version used in this paper, related to STAR Methods.

Version name	Strain number	Comment	Details
yeast_chr07_3_56	NA	Original design sequence	Final design by BioStudio
yeast_chr07_3_57	NA	Updated design sequence	Swapped two genes of TAG to TAA (*YGL226C-A* and *YGL087C*)
yeast_chr07_9_01	YSy100	synVII draft strain, with 1 TAG stop codons, 7 loxPsym sites missing (one site is the consequence of debugging, 5 sites locate wild-type region), 0 wild-type PCRTags, 42 point mutations causing amino acid changes, 4505 bp wild-type region from *YGR290W* to right telomere;	Missing loxPsym sites: 409978-410011, 768480-768513, 953939-953972, 955439-955472.<br>Remaining TAG stop codons: 639475 A->G<br>Point mutations that cause amino acid changes: 35647 G->A, 63955 T->C, 118823 G->A, 211372 A->T, 228617 G->A, 228865 G->A, 228978 A->G, 280790 A->T, 290392 C->T, 294107 G->A, 302039 C->T, 319501 G->A, 319502 G->A, 394086 T->C, 452503 A->G, 513232 A->G, 576521 T->A, 580950 A->C, 581229 A->G, 582191 T->C, 586977 A->T, 591572 T->C, 601536 T->C, 602470 G->A, 638403 T->A, 638759 A->G, 640573 A->G, 640791 A->G, 641509 A->C, 642293 T->G, 662375 A->T, 666764 T->C, 671909 ATT->A, 692026 C->A, 693273 A->G, 694871 T->C, 697270 T->C, 697348 A->G, 747229 C->T, 773455 C->G, 787845 G->T, 992161 A->G<br><br>Wild-type region between 1024448 and 1028925
yeast_chr07_9_02	YSy101	synVII draft strain, with 1 TAG stop codons, 11 loxPsym sites missing (one site is the consequence of debugging, nine sites locate wild-type region), 18 wild-type PCRTags (locate wild-type region), 42 point mutations causing amino acid changes, 12563 bp wild-type region from *YGR252W* to	Missing loxPsym sites: 409978-410011, 768480-768513, 945933-945966, 951991-952024, 953939-953972, 955439-955472, 1024448-1024481, 1024833-1024866, 1026944-1026977, 1028314-1028347, 1028616-1028649.<br>Remaining TAG stop codons: 639475 A->G<br>Wild-type PCRTags: 945146-945173 YGR252W_1_synF, 945590-945617 YGR252W_1_synR, 946550-946577 YGR253C_1_synF, 946742-946769 YGR253C_1_synR, 948986-949013 YGR254W_1_synF, 949238-949265 YGR254W_1_synR, 950922-950949 YGR255C_1_synF, 951183-951210 YGR255C_1_synR, 952735-952756 YGR256W_1_synF, 952927-952954 YGR256W_1_synR, 954240-954267 YGR257C_1_synF, 954705-954732 YGR257C_1_synR, 955686-955713 YGR258C_1_synF, 955950-955977 YGR258C_1_synR, 956349-956376 YGR258C_2_synF, 956796-956823 YGR258C_2_synR, 957492-957519 YGR258C_3_synF, 957681-957708 YGR258C_3_synR<br><br>Point mutations that cause amino acid changes: 35647 G->A, 63955 T->C, 118823 G->A, 211372 A->T, 228617 G->A, 228865 G->A, 228978 A->G,

		YGR258C, 4505 bp wild-type region from *YGR290W* to right telomere	280790 A->T, 290392 C->T, 294107 G->A, 302039 C->T, 319501 G->A, 319502 G->A, 394086 T->C, 452503 A->G, 513232 A->G, 576521 T->A, 580950 A->C, 581229 A->G, 582191 T->C, 586977 A->T, 591572 T->C, 601536 T->C, 602470 G->A, 638403 T->A, 638759 A->G, 640573 A->G, 640791 A->G, 641509 A->C, 642293 T->G, 662375 A->T, 666764 T->C, 671909 ATT->A, 692026 C->A, 693273 A->G, 694871 T->C, 697270 T->C, 697348 A->G, 747229 C->T, 773455 C->G, 787845 G->T, 992161 A->G<br><br>Wild-type region: 945146-957708 ,1024448-1028925
yeast_ chr07 _9_03	YSy105	Repaired 4505 bp wild-type region from *YGR290W* to right telomere, Repaired wild-type W region from *YGR254W* to *YGR246C* and *tN(GUU)G* gene was replaced back in yeast_chr07_9_02	Missing loxPsym sites: 409978-410011, 768480-768513, 953939-953972, 955439-955472.<br>Remaining TAG stop codons: 639475 A->G.<br>Wild-type PCRTags: 659213-659240 *YGR099W*_1_synF, 659567-659594 *YGR099W*_1_synR, 954240-954267 *YGR257C*_1_synF, 954705-954732 *YGR257C*_1_synR, 955686-955713 *YGR258C*_1_synF, 955950-955977 *YGR258C*_1_synR.<br>Point mutations that cause amino acid changes: 35647 G->A, 63955 T->C, 118823 G->A, 211372 A->T, 228617 G->A, 228865 G->A, 228978 A->G, 280790 A->T, 290392 C->T, 294107 G->A, 302039 C->T, 319501 G->A, 319502 G->A, 394086 T->C, 452503 A->G, 513232 A->G, 576521 T->A, 580950 A->C, 581229 A->G, 582191 T->C, 586977 A->T, 591572 T->C, 601536 T->C, 602470 G->A, 606435 A->AT, 606442 C->T, 638403 T->A, 638759 A->G, 640573 A->G, 640791 A->G, 641509 A->C, 642293 T->G, 728595 GA->G, 747229 C->T, 773455 C->G, 787845 G->T, 956885 A->G, 992161 A->G<br>deletion of *tN(GUU)G* at 694364 was replaced by the native sequence.
