# Supplementary material for: Dissecting aneuploidy phenotypes by constructing Sc2.0 chromosome VII and SCRaMbLEing synthetic disomic yeast
Source: Cell Genom. 2023 Nov 9;3(11):100364. doi: 10.1016/j.xgen.2023.100364 (PMC10667312; doi:10.1016/j.xgen.2023.100364)
Supplement: Document S2. Article plus supplemental information [file mmc3.pdf]

Cell Genomics

Article

Dissecting aneuploidy phenotypes by constructing Sc2.0 chromosome VII and SCRaMbLEing synthetic disomic yeast

Graphical abstract

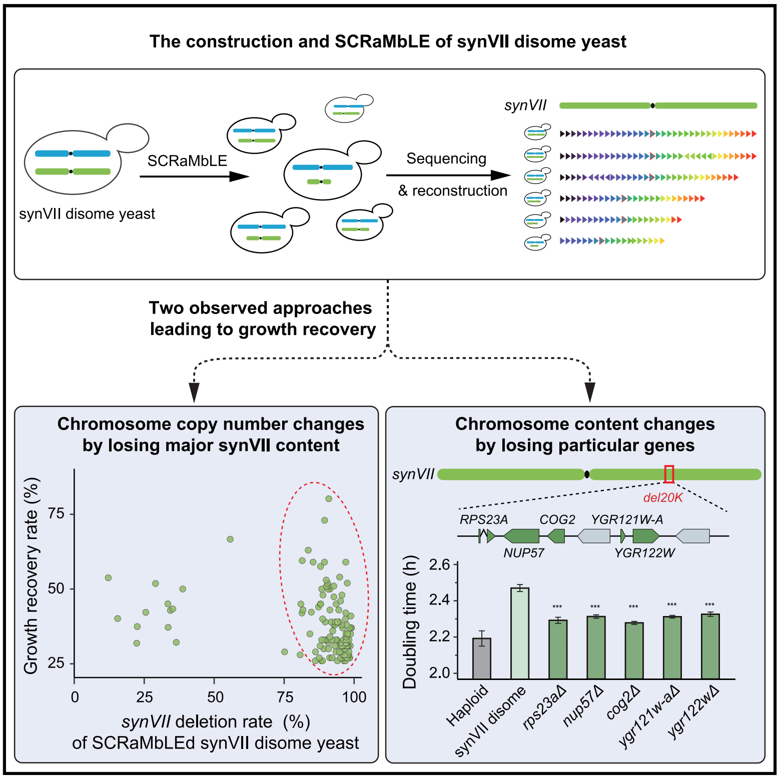

Highlights

- *De novo* design and synthesis of a Mb-scale synthetic yeast chromosome VII
- A disomic yeast (n + *synVII*) is constructed for dissecting the aneuploidy phenotype
- Chromosomal copy number and content both contribute to aneuploidy phenotypes
- Five genes on *synVII* contribute to alleviating aneuploidy phenotype

Authors

Yue Shen, Feng Gao, Yun Wang, ..., Huanming Yang, Xun Xu, Yizhi Cai

Correspondence

shenyue@genomics.cn (Y.S.),
yizhi.cai@manchester.ac.uk (Y.C.)

In brief

By construction and SCRaMbLE of a synthetic disomic yeast (n + *synVII*), Shen et al. discovered that both chromosome copy number and specific chromosomal regions (five genes on *synVII*) contribute to the aneuploidy-related phenotypes.

Shen et al., 2023, Cell Genomics *3*, 100364
November 8, 2023 © 2023 The Authors.
https://doi.org/10.1016/j.xgen.2023.100364

CellPress

Cell Genomics

CellPress
OPEN ACCESS

Article

Dissecting aneuploidy phenotypes by constructing Sc2.0 chromosome VII and SCRaMbLEing synthetic disomic yeast

Yue Shen,[1,2,3,4,16,17,*] Feng Gao,[1,3,16] Yun Wang,[1,2,3,12,16] Yuerong Wang,[1,3,4,16] Ju Zheng,[1,3] Jianhui Gong,[1] Jintao Zhang,[1] Zhouqing Luo,[5,6,7] Daniel Schindler,[8,9] Yang Deng,[1] Weichao Ding,[1,3,4] Tao Lin,[1,3] Reem Swidah,[8] Hongcui Zhao,[1,2,3] Shuangying Jiang,[5,6] Cheng Zeng,[5,6] Shihong Chen,[1] Tai Chen,[1,2,3] Yong Wang,[1] Yisha Luo,[8] Leslie Mitchell,[10] Joel S. Bader,[11] Guojie Zhang,[12] Xia Shen,[13,14] Jian Wang,[1] Xian Fu,[1,2,3] Junbiao Dai,[5,6] Jef D. Boeke,[10,15] Huanming Yang,[1] Xun Xu,[1,2,3] and Yizhi Cai[8,*]

[1]BGI Research, Shenzhen 518083, China
[2]BGI Research, Changzhou 213299, China
[3]Guangdong Provincial Key Laboratory of Genome Read and Write, BGI-Shenzhen, Shenzhen 518120, China
[4]College of Life Sciences, University of Chinese Academy of Sciences, Beijing 100049, China
[5]Guangdong Provincial Key Laboratory of Synthetic Genomics, Shenzhen Key Laboratory of Synthetic Genomics, Center for Synthetic Genomics, Shenzhen Institute of Synthetic Biology, Shenzhen Institutes of Advanced Technology, Chinese Academy of Sciences, Shenzhen 518055, China
[6]College of Life Sciences and Oceanography, Shenzhen University, Shenzhen 518055, China
[7]State Key Laboratory of Cellular Stress Biology, School of Life Sciences, Faculty of Medicine and Life Sciences, Xiamen University, Xiamen 361102, China
[8]Manchester Institute of Biotechnology, University of Manchester, 131 Princess Street, Manchester M1 7DN, UK
[9]Max Planck Institute for Terrestrial Microbiology, Karl-von-Frisch-Strasse 10, 35043 Marburg, Germany
[10]Institute for Systems Genetics and Department of Biochemistry and Molecular Pharmacology, NYU Langone Health, New York, NY 10016, USA
[11]Department of Biomedical Engineering, Johns Hopkins University, Baltimore, MD, USA
[12]University of Copenhagen, Universitetsparken 15, 2100 Copenhagen, Denmark
[13]Greater Bay Area Institute of Precision Medicine (Guangzhou), Fudan University, Guangzhou, China
[14]Center for Global Health Research, Usher Institute, University of Edinburgh, Edinburgh, UK
[15]Department of Biomedical Engineering, NYU Tandon School of Engineering, Brooklyn, NY 11201, USA
[16]These authors contributed equally
[17]Lead contact
*Correspondence: shenyue@genomics.cn (Y.S.), yizhi.cai@manchester.ac.uk (Y.C.)
https://doi.org/10.1016/j.xgen.2023.100364

SUMMARY

Aneuploidy compromises genomic stability, often leading to embryo inviability, and is frequently associated with tumorigenesis and aging. Different aneuploid chromosome stoichiometries lead to distinct transcriptomic and phenotypic changes, making it helpful to study aneuploidy in tightly controlled genetic backgrounds. By deploying the engineered SCRaMbLE (synthetic chromosome rearrangement and modification by loxP-mediated evolution) system to the newly synthesized megabase Sc2.0 chromosome VII (*synVII*), we constructed a synthetic disomic yeast and screened hundreds of SCRaMbLEd derivatives with diverse chromosomal rearrangements. Phenotypic characterization and multi-omics analysis revealed that fitness defects associated with aneuploidy could be restored by (1) removing most of the chromosome content or (2) modifying specific regions in the duplicated chromosome. These findings indicate that both chromosome copy number and specific chromosomal regions contribute to the aneuploidy-related phenotypes, and the synthetic chromosome resource opens new paradigms in studying aneuploidy.

INTRODUCTION

Aneuploidy represents an imbalanced genomic state, in which the copy number of intact or partial chromosomes is altered. At the organismal level, aneuploidy in humans is often intrinsically linked to embryonic lethality, particularly in early development with major developmental abnormalities, devastating genetic disorders, tumorigenesis, and aging.[1–5] Therefore, systematic investigation of the underlying molecular mechanisms of aneuploidy is essential to unravel its effects on basic cellular and developmental functions, as well as its clinical relevance as a prognostic marker or potential therapeutic target.

Early studies of aneuploid yeast, mouse, and human cells have unveiled a number of phenotypes and distinct gene expression

This is an open access article under the CC BY license (http://creativecommons.org/licenses/by/4.0/).

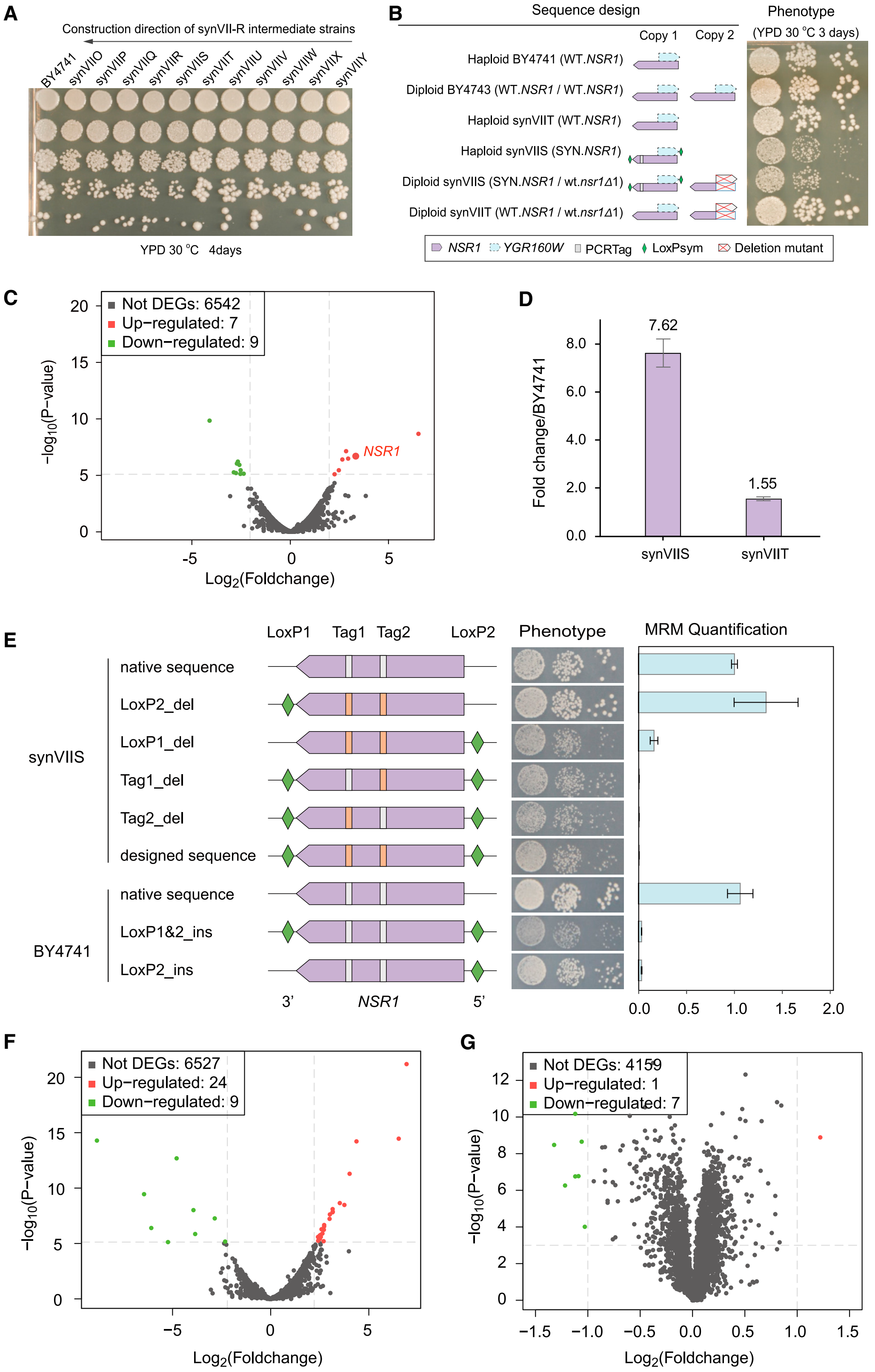

(legend on next page)

patterns. Different possible mechanisms have been proposed, attributing the aneuploid phenotypes to changes in many or a small number of critical genes ("mass action of genes" or "few critical genes" hypotheses),[6,7] resulting in stoichiometric imbalances between different subunits of cellular protein complexes.[8–12]

Although substantial efforts have been devoted to elucidating the causes and consequences of aneuploidy in recent years, the molecular mechanisms underlying the diverse aneuploid phenotypes remain poorly understood. The difficulty in identifying links between aneuploidy and its associated distinct phenotypes mainly derives from two reasons: (1) the consequences of aneuploidy vary significantly in the context of distinct karyotypes and cell types, and (2) limitations of current methods to generate isogenic and stable aneuploid cell populations in multi-cellular organisms.[2] Thus, as a unicellular eukaryote, the budding yeast *Saccharomyces cerevisiae* is widely adopted as a simple and suitable model for studying aneuploidy.[13] A method has been established to generate aneuploid yeast strains with defined karyotypes by induction of mis-segregation of target chromosomes during mitosis.[14] However, this method allows only the investigation of immediate consequences of karyotypic changes but fails to further identify the effects of specific regions within a chromosome.

In recent years, DNA synthesis and editing technologies have rapidly evolved and propelled synthetic genomics to center stage,[15] best exemplified by the Sc2.0 project that generated a series of synthetic yeast strains bearing designer chromosomes synthesized from scratch.[16–21] As a unique feature of the Sc2.0 genome, the SCRaMbLE (synthetic chromosome rearrangement and modification by loxP-mediated evolution) system allows the generation of combinatorial genomic diversity through massive rearrangements between designed recombinase recognition sites. This capability has been harnessed for applications including strain improvements for product yield and specific stress tolerance.[22–28] This on-demand genome rearrangement feature also makes Sc2.0 synthetic yeast a superior model for dissecting the complexity underlying cellular aneuploidy. By generating isogenic aneuploid yeast strains bearing defined Sc2.0 designer chromosomes, SCRaMbLE will enable generating diverse chromosomal rearrangements specifically on the synthetic chromosome(s). Combined genomic, transcriptomic, proteomic, and karyotype analyses of the resultant aneuploid yeast strains will shed new light on genotype-to-phenotype relationships associated with aneuploidy.

In this study, we construct the Sc2.0 chromosome VII (*synVII*) and its corresponding disomic yeast to demonstrate the feasibility of this approach. *SynVII* was chosen for two main reasons: first, because the cost of aneuploidy is reported to be proportional to the chromosome length,[29] *synVII* is one of the largest chromosomes at over 1 million base pairs, representing nearly 10% of the whole yeast genome. Second, few studies conducted in-depth analysis of the cause and consequence of disomic yeast bearing an extra copy of chromosome VII (*chrVII*).[30] Therefore, our study could facilitate the investigation of multiple aspects of aneuploidy using the disomic synVII yeast as a model system. We identify 219 SCRaMbLEd disomic yeasts with massive chromosomal rearrangements specifically limited to *synVII*. Phenotypic characterization and multi-omics analyses reveal two distinct approaches adopted by aneuploid yeast to restore cellular fitness. The substantial fitness cost as the result of aneuploidy can be restored by removing the majority of content from the additional chromosome copy. Interestingly, we found the deletion of a 20-kb region on the right arm of *synVII* is associated with upregulation of translation and leads to fitness improvement in varying conditions. Our results indicate that both chromosomal copy number and specific gene content contribute to the aneuploidy phenotypes.

RESULTS

The debugged synVII strain exhibits high fitness comparable with the wild-type strain

To utilize SCRaMbLE of synthetic disomic yeast for dissecting aneuploidy phenotypes, we started with the construction of synthetic *chrVII*. *SynVII* was designed following the previously reported Sc2.0 design principles,[31] resulting in a final 1,028,952-bp synthetic chromosome carrying ~11.89% modified sequence in comparison with the native sequence.

Three unexpected design features causing significant fitness defects were identified and corrected during the construction process. The intermediate strain containing synthetic megachunkcS-Y (synVIIS) revealed a significant growth defect compared with the parental strain synVIIT (because the chromosome was constructed from "right" to "left") and wild-type (WT) strain BY4741 in rich medium (yeast extract peptone dextrose [YPD]) at 30°C (Figure 1A). By mating the synVIIS strain (*MAT*α) with all 25 single-gene knockout strains[32] corresponding to the megachunk S region, we found that the synthetic *NSR1* gene led to the observed fitness defect (Figure 1B). Notably, transcriptome profiling of the synVIIS strain revealed an ~6-fold

Figure 1. Functional dissection and repair of SynVII

(A) Spot assay of synVII-R intermediate strains. The synVII-R is constructed in orientation from megachunk Y toward megachunk O.

(B) Bug mapping by mating growth-defective query strain synVIIS and its parental strain synVIIT with yeast single-knockout *nsr1Δ1* strain.

(C) Transcriptome profiling of synVIIS compared with synVIIT reveals significant upregulation of *NSR1* mRNA. Upregulated features are labeled in red, and downregulated features are labeled in green.

(D) Quantitative real-time PCR (qPCR) validation of *NSR1* mRNA expression in synVIIS and synVIIT strains. Error bars represent ±SD from three independent experiments.

(E–G) Introduction of loxPsym (green) at the 5′ UTR of NSR1 led to a growth defect in both synVII intermediate strain synVIIS and wild-type strain BY4741. The corresponding phenotype by plating and protein expression level, quantified by multiple reaction monitoring mass spectrometry (MRM-MS) analysis, is shown for each constructed strain. Error bars indicate ±SD (n = 3). The white and orange blocks represent wild-type and synthetic PCRTags, respectively. Identified dysregulated genetic features at (F) transcriptome level and (G) proteome level of repaired synVII cells compared with BY4741. Total number of differentially expressed ($p < 0.001$) features in transcriptome and proteome are presented.

upregulation of *NSR1* transcription compared with synVIIT, which was further confirmed by quantitative reverse transcription PCR (RT-qPCR) analysis (Figures 1C and 1D), whereas Nsr1 protein abundance was drastically reduced paradoxically (Figure 1E). There are several design features of the *NSR1* gene: two synthetic PCRTags within the coding region and one loxPsym site at the 3′ UTR were introduced into synthetic *NSR1*; in addition, the *YGR160W* "dubious ORF" overlapped the *NSR1* gene on its complementary strand, resulting in the insertion of an additional loxPsym site immediately downstream of its stop codon, as dictated by the "rules" of the Sc2.0 design. This resulted in one loxPsym site within the 5′ UTR of *NSR1* (Figure 1B). We hypothesized that this "misplaced" loxPsym site might be responsible for the fitness defect. To this end, we individually reverted each designer feature in or near *NSR1* to the WT counterpart and monitored growth and *NSR1* expression. The steady-state level of *NSR1* returned to the WT level by simply removing the loxPsym site at the 5′ UTR of *NSR1* (Figure 1E). In comparison, the removal of the synthetic PCRTags had little effect. These results demonstrate that the loxPsym site at the 5′ UTR of the *NSR1* gene leads to the fitness defect, presumably by interfering with translation because the *loxP* sequence can form a stem-loop structure.[33] This interpretation accounts for the paradox described previously, namely, increased *NSR1* mRNA abundance (presumably a consequence of the reduced protein expression level). Consistent with this finding, the introduction of a loxPsym sequence at the 5′ UTR of *NSR1* resulted in dramatic decreases in protein level and obvious growth defect in the BY4741 strain (Figure 1E). A similar pattern was also observed in a synX bug. One loxPsym site was "accidentally" transcribed as a part of *SWI3* 5′ UTR, which led to an increased transcript but reduced protein level.[34] In addition, we noticed that the removal of loxPsym site at the 3′ UTR could alleviate the cellular defect in the WT strain to some extent, suggesting the formation of a potential stem-loop by the two loxPsym sites could further affect the translation. Taken together, significant fitness defect observed in the intermediate synVIIS strain was derived from the loxPsym site inserted into the UTR of *NSR1*. Another two fitness defects caused by the loxPsym site in megachunk W (Figure S1A) and the removal of the tRNA *tN(GUU)G* gene (Figure S1B) were identified and corrected, followed by verification of whole-genome sequencing, phenotypic, transcriptomic, and proteomic profiling in comparison with the parental strain BY4741. Our results demonstrate that the final version of the synthetic chromosome has a minimal effect on yeast cell physiology and transcriptional and translational states (Figures 1F, 1G, and S2). Therefore, the synVII strain with its in-depth characterized phenotype is ideal for further aneuploidy studies.

The *synVII* chromosome in disomic yeast is well maintained and leads to aneuploidy-specific phenotypes

To build a disomic yeast strain with a defined chromosome gain, we took advantage of a well-established system to generate a *chrVII*-specific aneuploid yeast strain.[14,35] The galactose inducible/glucose repressible *GAL1* promoter was inserted adjacent to the *CEN7* sequence of WT strain BY4742 to allow controlled inactivation of centromere function, which led to transient nondisjunction of *chrVII* and the generation of the yeast strain YSy140 with two copies of native *chrVII*. YSy140 (*MATα*) and synVII strain YSy105 (*MAT***a**) were mated and sporulated to obtain the disomic yeast YSy142 bearing one synthetic and one native copy of *chrVII* (Figure 2A), which was verified by the whole-genome sequencing.

Next, we systematically examined the consequences of gaining a synthetic *chrVII* in the disome strain YSy142 through detailed analyses of chromosome organization, transcriptional profile, genome stability, and phenotypes. By investigating the trajectories of both synthetic and WT *chrVII* of YSy142, we found that the two chromosomes are symmetrically arranged in the nucleus (Figure 2B). Transcriptome profiling revealed that the majority of genes present on both copies of *chrVII* in YSy142 and YSy140 strains were transcribed (Figure 2C). In the 563 genes on *chrVII*, 517 and 540 genes were upregulated in YSy140 and YSy142, respectively, in comparison with BY4741. Despite the presence of an extra copy of *chrVII*, only less than 10% of genes exhibited a buffered expression level. Previous studies have described a common aneuploidy gene-expression (CAGE) signature in aneuploid yeast-cell populations.[12] We found YSy140 and YSy142 strains exhibited a similar aneuploidy gene-expression profiling according to the CAGE patterns with a highly consistent rate (Figure S3). These results suggested that the disome strains YSy140 and YSy142 shared a common aneuploidy transcriptomic profiling and cell physiology.

Previous studies have shown that some aneuploid strains are unstable.[36] Here we analyzed the stability of YSy142 and YSy140 through long-term growth assays (~220 generations). Compared with YSy140, YSy142 showed a significant improvement of genome stability. More than 50% of the population of YSy142 stably maintained the *synVII* chromosome over 160 generations. In comparison, the aneuploid strain YSy140 was stable only up to ~80 generations (Figure 2D). The integrity of target chromosomes in both YSy142 and YSy140 was confirmed by whole-genome sequencing (Figure S4). Overall, the stability of an extra copy of synthetic *chrVII* in the YSy142 strain provides an unprecedented opportunity to dissect *chrVII*-specific aneuploidy-associated molecular and phenotypic changes.

To determine how aneuploidy affects the proliferation and physiology, we further characterized aneuploid synVII strains YSy140 and YSy142 under conditions with different types of exogenous stress and identified two aneuploidy-specific phenotypes (Figure S5). Specifically, disomic strains exhibited increased sensitivity to cycloheximide (a protein synthesis inhibitor) and hydroxyurea (an inhibitor of ribonucleotide reductase) and methyl methanesulfonate (MMS; a DNA-damaging agent). These findings are consistent with traits shared by most aneuploid yeast strains harboring different karyotypes reported in previous studies.[11,37]

High-frequency rearrangements revealed in 219 SCRaMbLEd disomic yeasts with varying degrees of growth recovery

Previous studies demonstrated that extensive unique genotypes could be generated by SCRaMbLE of synthetic chromosomes within populations of Sc2.0 synthetic strains.[22–25,28,38] With the

CellPress
OPEN ACCESS

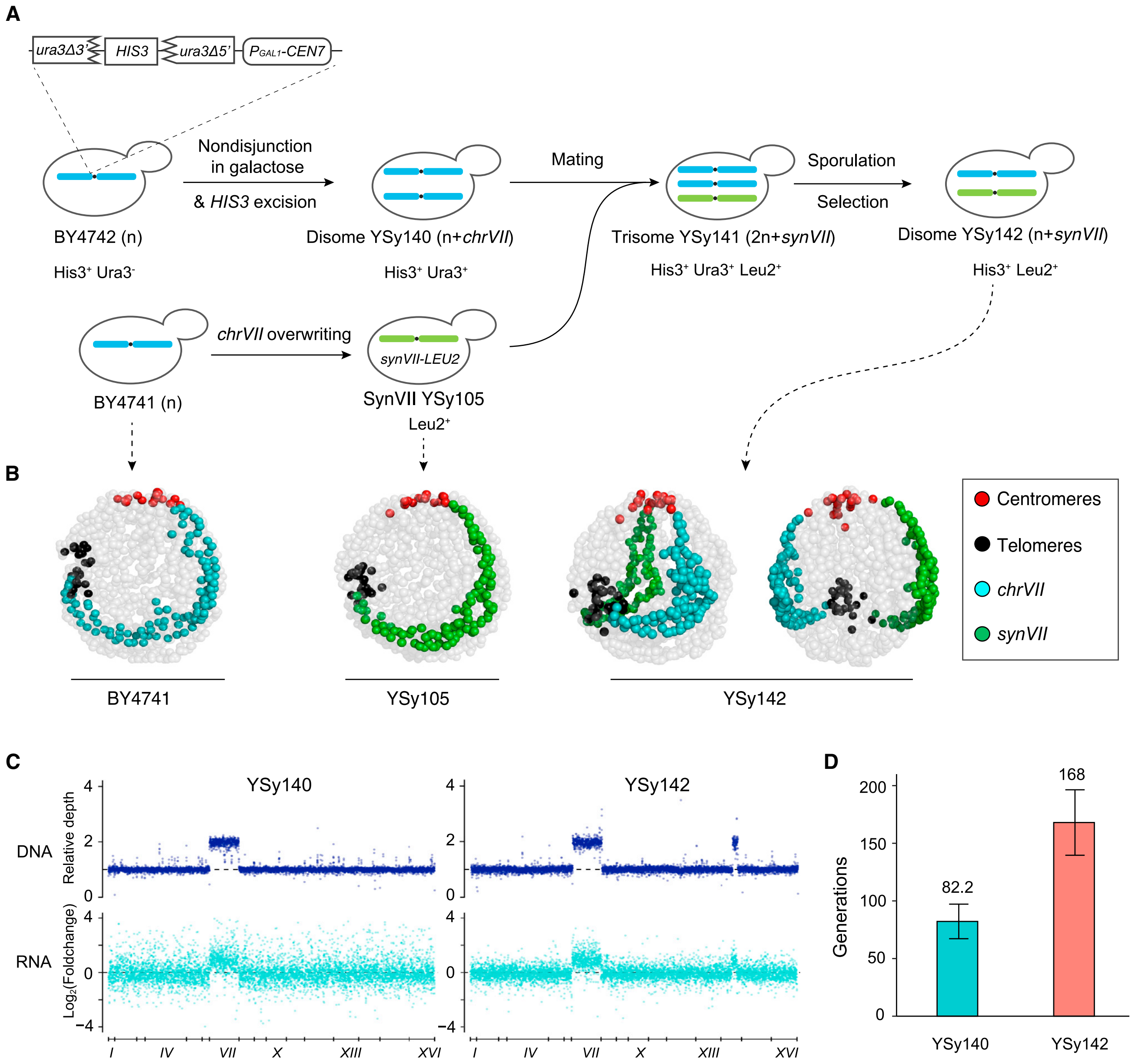

Figure 2. Construction and physiologic analysis of disomic yeasts

(A) Schematic illustration of the construction of disome yeast strains YSy140 and YSy142.

(B) 3D genome organization of native and synthetic chromosome VII in YSy142 strain in comparison with haploid BY4741 and YSy105. Each bead represents a 10-kb chromosome segment. Centromeres, telomeres, and wild-type *chrVII* and *synVII* are indicated with red, black, blue, and green beads, respectively. Other chromosomes are shown in gray. Two angles of view are shown for the YSy142 strain.

(C) The corresponding DNA and mRNA levels track with gene copy number in both disomic yeast strains YSy140 and YSy142. A 60-kb tandem duplication in chromosome XIV is identified in YSy142 and all derived SCRaMbLEd strains.

(D) Genome stability analysis of YSy140 and YSy142 through long-term growth assays across a time span of ~220 generations. The number represents the average number of generations maintaining aneuploidy. Error bars indicate ±SD (n = 3).

See also Figure S4.

constructed aneuploid yeast strain harboring a complete *synVII*, we sought to determine whether SCRaMbLE could help screen for aneuploid cells, which recovered a WT phenotype, offering us an opportunity to identify the genes/target regions that drive the aneuploidy-specific fitness defects. To this end, a daughter-cell-specific Cre recombinase expression plasmid, pSCW11-*creEBD*,[39] was transformed into YSy142 strain to promote SCRaMbLE. After 24 h, cultured cells were plated onto selective agar medium containing translation inhibitor cycloheximide to select for SCRaMbLEd aneuploid synVII derivatives.

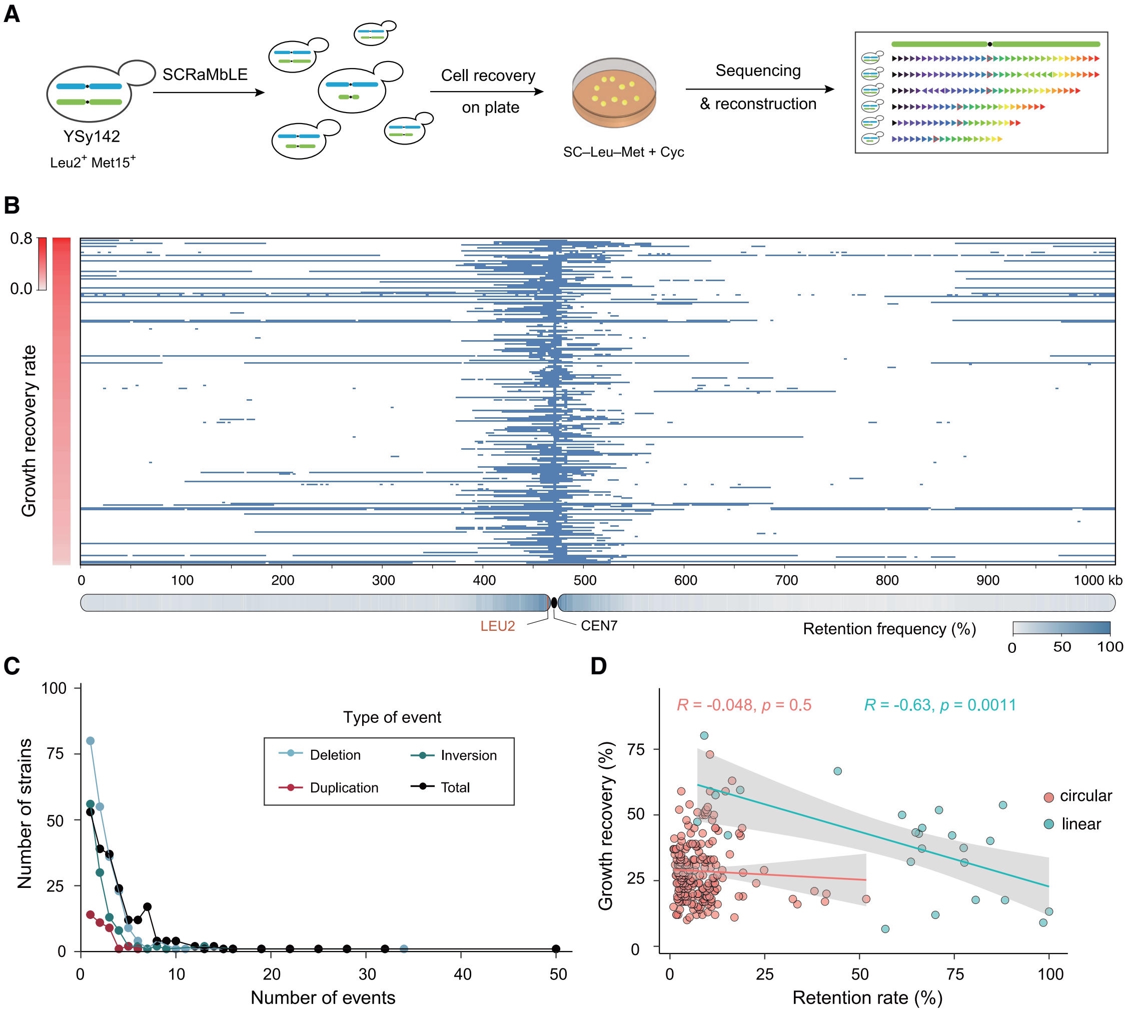

Figure 3. SCRaMbLE of YSy142 disomic yeast

(A) SCRaMbLE and analysis workflow. The auxotrophic marker of the YSy142 strain was swapped from *HIS3* to *MET15*. See construction of aneuploid synVII strain section in the STAR Methods for details.

(B) The fate of each segment flanked by two loxPsym sites in each strain is indicated as preserved (light blue) or deleted (white) by any SCRaMbLE event. The y axis shows the relative average phenotypic recovery rate of each SCRaMbLEd strain in comparison with that of YSy142 represented by the color scale (n $\geq$ 200).

(C) The distribution of recombination events for all selected SCRaMbLEd aneuploid strains. The number of events per strain has a long tail with some strains having 22 and 25 distinct recombination events.

(D) Correlation between chromosome retention rate and growth recovery in SCRaMbLEd strains with circular *synVII* and SCRaMbLEd strains with linear *synVII*. Each dot represents one SCRaMbLEd strain. R indicates Pearson's correlation. Solid line: fitted curve (ggplot:geom_point), geom_smooth (method = lm, se = TRUE). Gray area: 95% confidence range for fitted curve.

The dual auxotrophic selection (*Leu2*$^{+}$*Met*$^{+}$) of target chromosomes was utilized to maintain the aneuploidy state and ensure that the phenotype is not due to the simple loss of one chromosome copy. The SCRaMbLEd colonies showing improved growth in the presence of cycloheximide were analyzed by genome sequencing (Figure 3A).

In all 219 selected SCRaMbLEd strains, the growth recovery rate was calculated by comparing average colony size (from $\geq$200 single colonies) of each strain with that of the un-SCRaMbLEd aneuploid yeast parent YSy142. Our results revealed varying degrees of growth recovery rate from 6.6% to 80% for SCRaMbLEd strains harboring distinct content and size of *synVII* (Figure 3B). Not surprisingly, a peak of sequence retention was found within *CEN7*-adjacent regions because of the maintenance of selection for prototrophy (*Leu2*$^{+}$). However, from the whole-genome sequencing, we observed an unequal proportion of reads of WT versus synthetic *synVII* PCRTags for three SCRaMbLEd strains, with the relative ratio at around 2:1 (Figure S6A). Presumably, the ratio should be equally distributed because there is one copy of WT and synthetic *chrVII* each in the disome yeast. Further analysis by flow cytometry on one of the three strains confirmed that it is a trisomic yeast (Figure S6B), possibly resulting from spontaneous whole-genome duplication (WGD) after or during SCRaMbLE. Thus, these three strains are

excluded from the collection of selected SCRaMbLEd strains for further analysis because their phenotypes might also be influenced by the corresponding genome ploidy, as suggested in previous studies.[37]

Among the 969 recombination events that occurred in cells that showed recovery of cellular fitness, three types of event, including deletion, inversion, and duplication, were observed at frequencies of 62.2%, 29.2%, and 8.6%, respectively. A recent SCRaMbLE study suggested that the recombination frequency in a haploid synthetic yeast was not as high as that in the right arm of synthetic chromosome IX (*synIXR*) experiment.[24] It is very likely that the random recombination between two loxPsym sites in a region containing essential genes in haploid yeast would lead to lethality and consequently result in a bias of recombination events, although the random nature of SCRaMbLE events is preserved in heterozygous diploids.[26] Presumably, in our study, we might observe an increase of recombination frequency because SCRaMbLE occurs only in the synthetic chromosome, whereas the native copy remains intact. For each SCRaMbLEd strain, we observed an average number of events at 4.42. We also observed a very long tail in the distribution of events per strain, with ≥ 10 events found in 17 strains, and 2 strains containing 32 and 50 events, respectively (Figure 3C).

In general, we observed a negative correlation between chromosome retention rate (the frequency of each *synVII* segment preserved after SCRaMbLE across all selected SCRaMbLEd strains) and growth recovery rate (the relative colony size of each SCRaMbLEd strain compared with YSy142 under the same culturing condition) (Figure 3D). We found 31 SCRaMbLEd strains carrying circular chromosomes with higher growth recovery rates (40%–60%) tended to lose most of both *synVII* chromosome arms, retaining only 1%–19% of the original chromosome arms, namely, the centromere-adjacent regions. In contrast, 18 of the 24 strains with >50% retention rate showed only moderate growth recovery rates (averaging 32.4%). Our result supports the idea that gene dosage contributes heavily to aneuploidy and by reducing the copy number via SCRaMbLE, the growth defect of disomic yeast is rescued.

The frequency of circular *synVII* is significantly higher than that of linear *synVII* maintained in SCRaMbLEd disomic strains

We identified two types of *synVII* chromosome structural conformation (circular and linear forms) in the 219 SCRaMbLEd disomic yeast. Interestingly, the frequency of generating circular SCRaMbLEd synthetic chrVII was surprisingly high. Around 89% of all selected SCRaMbLEd disomic strains (in total 195) maintained circular SCRaMbLEd *synVII* with sizes ranging from 10 to 532 kb, whereas only 11% of selected strains (in total 24) carried the original linear SCRaMbLEd *synVII* with sizes ranging from 74 to 1,028 kb. One possible explanation for this is that once a circularized chromosome forms, which requires a single intramolecular SCRaMbLE event between *loxP* sites near the two telomeres, it is "primed" to give rise to subsequent daughter deletions that can remove most of both chromosome arms. Moreover, whereas linear *synVII* can continually give rise to additional daughter circles, once locked into the circular state it cannot return to a linear state via SCRaMbLE. Another potential explanation is that some (or several) genes near one of the telomeres are very toxic in multiple copies. Thus, the formation of circular chromosomes would remove this gene preferentially and may have been selected for.

We observed that the average coverage depth of the circular SCRaMbLEd *synVII* was approximately three times lower than the native copy (Figure S7). One theoretical explanation for this observation is that of WGD followed by loss of one copy of the synthetic chromosome, yielding a 2n+1 strain. This was ruled out by both flow cytometry measurement and sporulation analysis (Figure S8). It is possible that circular *synVII* is not stable and lost in a subpopulation, raising the concern that the improved fitness is due to the average lowered copy number in the mixed population. To exclude this possibility, we selected three representative samples with distinct circular *synVII* contents from the 195 strains for long-term genome stability assays. Our results showed that the genome of SCRaMbLEd aneuploid strains is fairly stable. More than 50% of the population stably maintains the circular SCRaMbLEd *synVII* chromosome over 60–200 generations in the absence of selection (Figure S9). Considering at the time ($\sim$3 days culturing) of phenotypic assays and sampling for genome sequencing, more than 90% of the population in each selected strain maintained the SCRaMbLEd *synVII* chromosome, we conclude that the apparently low copy number of circular SCRaMbLEd *synVII* is not the main reason for the observed growth recovery. It has been previously reported that the average read depth for the non-synthetic chromosome is greater than that for the synthetic circular chromosome arm, indicating higher recovery of linear versus circular chromosomes during the sample preparation process for sequencing,[22,39] potentially explaining the observed lower depth of circular SCRaMbLEd *synVII*.

In contrast with SCRaMbLEd strains bearing circular *synVII*, the recovery rates of screened strains that retained linear *synVII* exhibit a relatively wide range (from 6.6% to 80.2%), with an average recovery rate of around 39.1%. Using a previously established method,[22] we reconstructed the full SCRaMbLEd *synVII* chromosome sequence of all 24 screened strains (Figure S10A). In 22 of 24 strains with varying recovered phenotype, the left arm of *synVII* (*synVIIL*) was well maintained, showing few SCRaMbLE events, whereas the chromosome content of *synVIIR* across these strains changed drastically. The most frequently observed recombination event was deletion, at a frequency of 61.9%, followed by inversion at a frequency of 34.1%. We further explored the deletion distribution across the entire chromosome and observed a deletion hotspot in the right arm region of *synVII* (Figure S10B). For the top 18 strains with relatively high fitness improvement (average recovery rate >30%), we visualized the copy number of each segment between two adjacent loxPsym sites in the original order of *synVII*. Large fragment losses within the middle of the chromosome's right arm and some small deletions also located in the left arm represented the most common deletion types (Figure S10C).

The loss of major chromosome content by SCRaMbLE leads to significant fitness improvement of disomic yeast with reversed environmental stress response

Strains losing 85% of the chromosome content represent the leading type among all selected 195 SCRaMbLEd strains with

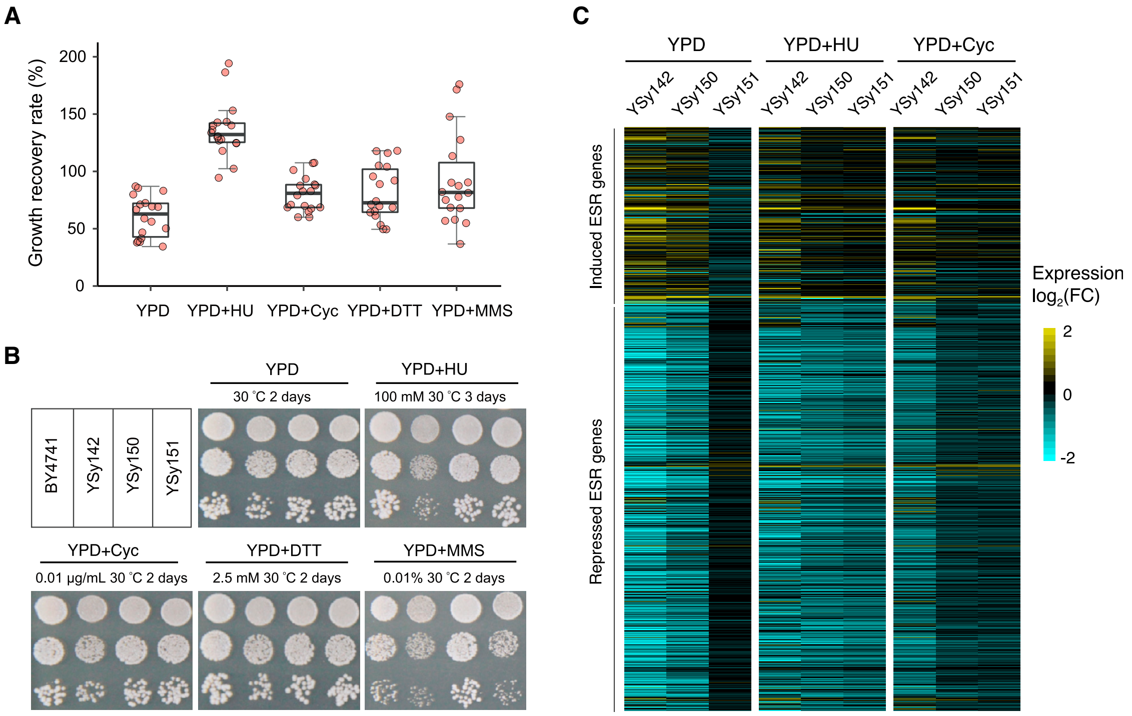

Figure 4. Growth assay and ESR profiling of disomic yeast with circular SCRaMbLEd *synVII*
(A) General improvement of fitness at varying degrees was observed for the top 18 SCRaMbLEd strains in five representative conditions. Each dot represents average growth recovery rate of one SCRaMbLEd strain calculated based on multiple single colonies (n ≥ 200).
(B) Spot assay of two representative strains of the 18 SCRaMbLEd strains under various conditions, showing general improved fitness.
(C) The ESR genes expression profile of the unSCRaMbLE strain YSy142 and two SCRaMbLEd strains (YSy151 and YSy150) after normalization with the reference wild-type strain BY4741 in three selected conditions. The fold change is represented by the color scale (yellow: upregulated; blue: downregulated). Conditions include: YPD at 30°C for 2 days; YPD + cycloheximide (Cyc; 0.01 μg/mL) at 30°C for 2 days; YPD + DL-dithiothreitol (DTT; 2.5 mM pretreatment for 1 h) for 2 days; YPD + hydroxyurea (HU; 100 mM) at 30°C for 4 days; YPD + methyl methanesulfonate (MMS; 0.01% v/v) at 30°C for 3 days.
YPD, yeast extract peptone dextrose.

circular *synVII*, at a frequency of 91% (in total 178 strains), but the recovery rates of these strains varied significantly. We speculate that the retained chromosome contents include favored gene combinations that restore fitness under specific stress conditions, and thus the fitness of strains with varying retained gene contents would differ. We then selected the top 18 strains with recovery rate above 46% and examined their phenotypes under normal conditions (YPD), as well as under stress conditions related to DNA replication and repair, translation, and osmolarity regulation. Compared with the parental YSy142 strain, we observed a general improvement of fitness in all 18 strains, but to varying degrees (Figure 4A).

Previous studies described that exponentially growing disomic yeast strains typically exhibited a gene expression pattern designated as yeast environmental stress response (ESR), which includes the upregulation of ~300 genes and the downregulation of ~600 genes (also known as the “induced (i) ESR” and “repressed (r)ESR,” respectively).[10,40] Our transcriptome analysis consistently revealed that the disomic YSy142 also exhibited the ESR transcriptional signature. To be more specific, >70% of the reported iESR genes were upregulated and >80% of the rESR genes were downregulated in YSy142. Because the ESR pattern is highly correlated with the fitness of disomic yeast strains under stressful conditions,[41] we hypothesized that the SCRaMbLEd strains with recovered fitness would show a reversed ESR pattern. The fitness of both YSy150 and YSy151 SCRaMbLEd strains, which lost 95.2% and 88.4% of synthetic *chrVII*, respectively, was significantly improved under most tested conditions compared with YSy142 (Figure 4B). Thus, these two strains were chosen for further transcriptome analysis with a focus on ESR-related genes. As expected, our results revealed a reversed trend of the transcriptional signature in both strains under three selected representative conditions, in which most of the iESR/rESR genes no longer showed significant up-/downregulation compared with YSy142 (Figure 4C). These results demonstrate that the degree of the ESR correlates well with the cell proliferation rate, and the SCRaMbLE process is efficient and effective to recover aneuploidy-specific fitness.

The deletion of a 20-kb region on *synVII* is linked to upregulation of translation and leads to improved growth

In contrast with SCRaMbLEd strains with circular *synVII*, the SCRaMbLEd strains bearing linear *synVII* maintained the majority of chromosome content (chromosome retention rate ranging from 7.2% to 100%) and showed a relatively minor fitness

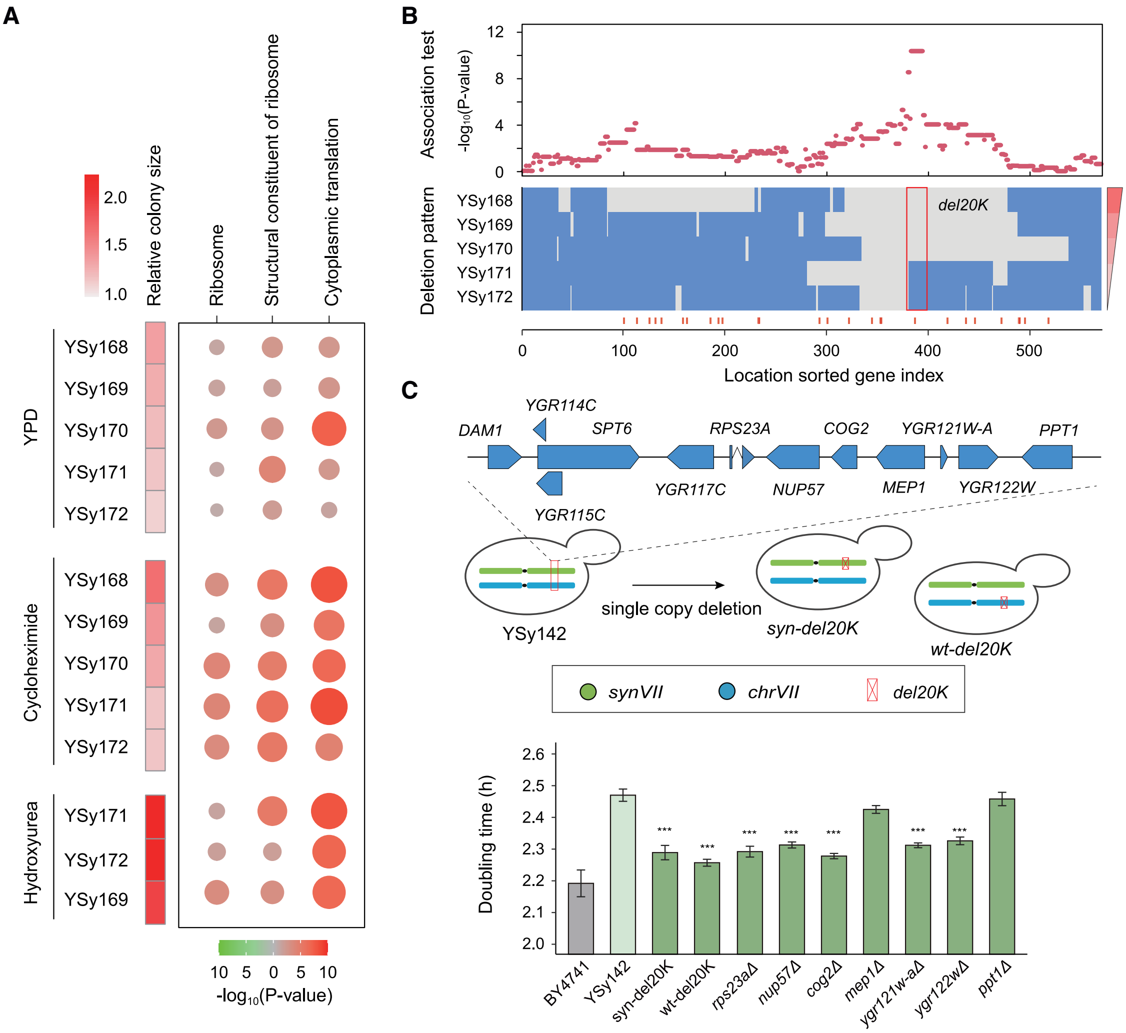

Figure 5. The improvement of *synVII* aneuploidy phenotypes by the deletion of a 20-kb region (del20K) on *synVIIR* is associated with the upregulation of protein biosynthesis

(A) The proteomics analysis of five disomic strains with linear SCRaMbLEd *synVII* exhibited varying recovery rates grown on YPD and two stress conditions.

(B) The chromosome-wide association analysis against the five disomic strains reveals a 20-kb region that might play an essential role in the observed fitness improvement. Each red dot represents the statistical significance of each gene for the association between its deletion pattern and relative colony size. The five selected strains are ranked by their corresponding growth recovery rate. The regions deleted and reserved after SCRaMbLE are presented in gray and blue, respectively.

(C) Doubling time measurement of YSy142, *syn-del20K*, *wt-del20K*, and YSy142 carrying the single-gene knockout strains under the Cyc condition. The error bar indicates standard error of the mean (SEM) in seven biological replicates. The p value was calculated using single-tailed Student's t test. ***p < 10E−3. YPD + Cyc (0.01 μg/mL) at 30°C; YPD + HU (100 mM) at 30°C.

See also Figures S10 and S11.

recovery rate. We aimed to disclose whether a distinct mechanism other than the "mass action of genes" hypothesis was utilized by these yeast cells to cope with the aneuploidy-induced stress. Thus, we performed comparative proteomics analysis on five disomic strains with linear SCRaMbLEd *synVII* that exhibited varying recovery rates grown under YPD and stress conditions containing cycloheximide and hydroxyurea (Figure 5A). In addition, we observed that protein synthesis and ribosome biogenesis processes were significantly upregulated in all selected strains under various conditions, suggesting that the increased activity of protein biosynthesis was linked to the improved growth of disomic strains.

Next, we wanted to determine the correlation among the observed rearrangement events on linear *synVII*, enhanced expression of the translation machinery, and improved phenotype. Based on the observed deletion hotspot on *synVII* and distribution of genes involved in translation and ribosome biogenesis, we looked for the key rearrangements that were potentially related to fitness improvement of disomic yeast strains after SCRaMbLE. We also performed a chromosome-wide association analysis to

study the recombination effect of every single gene on cell fitness and found that the deletion of several genes might be involved in fitness improvement (Figure 5B). In particular, a ∼20-kb deletion (del20K) in *synVII* showed the strongest association with growth recovery (p = 4.20E−11). We speculate that genes located in the del20K are likely responsible for the observed recovered phenotype of selected SCRaMbLEd strains. To further verify the causality of the del20K on growth recovery, we re-created the del20K deletion on *synVII* or WT chromosomes in YSy142 for further phenotypic analysis. By deleting one copy of either WT or synthetic del20K, we observed a notable improvement in cell growth (Figure 5C). This finding highlights the key role of del20K, a relatively modest structural variant, in rescuing *synVII* aneuploidy phenotypes. We further identified a ∼12-kb sub-region of the del20K, containing seven genes, as responsible for growth recovery (Figure S11). To further dissect the key genes, we individually removed the additional copy of these genes and found the deletion of five genes played positive roles in accelerating growth under both normal and stress conditions (Figures 5C and S11). Many of these effective genes encode known or putative regulators, including ribosomal protein Rps23a, nuclear pore component Nup57, Golgi complex component Cog2, potential PH regulation protein Ygr122w, and Ygr121w-a with uncharacterized function. Taken together, our results showed that the five genes in del20K might play a combinatorial role in alleviating *synVII* aneuploidy phenotypes. It will be of great interest to further dissect the mechanism of these genes involved in the del20K-dependent aneuploidy phenotypes.

DISCUSSION

Here we describe a novel system for studying the consequences of varying aneuploidy states in a controllable manner by SCRaMbLEing constructed aneuploid Sc2.0 yeast strain. In general, it is well-known that different aneuploid states can lead to distinct patterns of phenotypic and gene expression profiles and aneuploid cells bearing large chromosomal lesions often affecting hundreds of genes.[6,14,40,42] Following traditional approaches that are incapable of micro-manipulating the target chromosome content, it is extremely challenging, if not impossible, to systematically characterize the consequences and explore the causality of these phenotypes. In our study, we have demonstrated that this technical bottleneck was overcome by the developed inducible aneuploidy system, which enables controlled perturbation(s) of the specific chromosomal region(s) and has two advantages. Except for synthetic *chrVII*, the aneuploid strain YSy142 maintains the same genetic background as its parental haploid strains BY4741 and YSy105, thereby enabling the analysis of phenotypic consequences of aneuploidy in a consistent genetic background. In addition, the unique built-in SCRaMbLE design limits perturbation of the chromosomal structure and content to the synthetic chromosome, and consequently the investigation of the potential causal regions for aneuploid-specific phenotypes in a large chromosome of a defined karyotype is readily achieved.

We demonstrated the feasibility of using the disomic synVII strain YSy142 for studying aneuploidy, and we identified over 200 SCRaMbLEd aneuploid strains for in-depth analysis. Our result shows that losing the majority content of *synVII* chromosome can relieve the cell from growth defects under stress conditions, suggesting that "mass action" of genes across the entire *chrVII* is mainly responsible for the proliferation defect associated with aneuploidy. In addition, a 20-kb deletion hotspot on the middle half of the right arm of linear *synVII* chromosomes of SCRaMbLEd strains was associated with growth recovery. We further reconstructed this structural variant in disomic yeast strain YSy142 and demonstrated that the del20K suffices as responsible for the observed growth recovery. Our result suggests that in addition to gene dosage, cell growth rate can be accelerated by specific gene contents (only 2% of *chrVII* content in our case). By individually removing the additional copy of seven genes in the del20K region, we showed the genes of *RPS23A*, *NUP57*, *COG2*, *YGR121W-A*, and *YGR122W* could significantly alleviate the *synVII* aneuploidy phenotypes at levels of effect very close to del20K. It will be of great interest to further explore the mechanism of each gene's role or their combinatorial role involved in del20K-dependent aneuploidy phenotypes. Interestingly, we also found that the significant growth recovery for three SCRaMbLEd aneuploid strains was due to spontaneous WGD, followed by loss of one copy of *synVII*. With flow cytometry, we confirmed that these three SCRaMbLEd aneuploid strains that present near WT growth recovery have experienced a homozygous increase in the DNA content from disome (n+1) to 2n+1. It is known that yeast cells can undergo spontaneous alterations of cell ploidy to gain a growth advantage under stressful conditions.[43] Thus, we speculate that the observed spontaneous WGD was a compensatory response to the increased fitness burden triggered by aneuploidy; furthermore, it suggests that "dialing down" relative expression of the offending genes by half suffices to greatly reduce the fitness defect.

To summarize, the inducible aneuploidy system developed in this study holds great potential to be further applied to systematically construct a full 16-chromosome collection of aneuploid yeast strains heterozygous for synthetic chromosomes and to dissect the molecular mechanisms underlying how aneuploidy impacts cell physiology and disease states. In light of the recent completion of all Sc2.0 yeast chromosomes and the prospect of *de novo* assembly of chromosomes from other species, including humans, our strategy can potentially be applied to study aneuploidy in diverse chromosomal, cellular, and species contexts. With the technical feasibility of *de novo* designing and constructing megabase-scale chromosomes combined with the flexibility of genomic rearrangements conferred by SCRaMbLE, we envision our strategy will revolutionize synthetic genomics and aneuploidy studies.

Limitations of the study

There are still some present limitations and potential challenges of this study to be addressed in the future. First, the screening strategy used in this study is based on isolating SCRaMbLEd cells with recovered colony size after plating. A more efficient high-throughput screening strategy would be highly desirable to identify SCRaMbLEd strains with more diverse genotypes. Second, for the selected 219 SCRaMbLEd synVII disome yeasts with varying genotypes, the underlying mechanism responsible

for the observed growth rate recovery by particular structural variation(s) needs further in-depth investigation. Third, with the completion of more synthetic Sc2.0 chromosomes, the presence of inter-chromosome rearrangement between different synthetic chromosomes by SCRaMbLE will bring further challenges to the dissection of the potential correlation between genotype and aneuploidy-associated phenotypes.

CONSORTIA

This work is part of the international Synthetic Yeast Genome (Sc2.0) consortium. The chromosome design and building consortium includes research groups worldwide: Boeke lab at Johns Hopkins University and New York University (led chromosomes I, III, IV, VI, VIII, and IX), Chandrasegaran lab at Johns Hopkins (led chromosomes III and IX), Cai lab at University of Edinburgh and University of Manchester (led chromosomes II and VI and tRNA neochromosome), Yue Shen's team at BGI-Research SHENZHEN (led chromosomes II, VII, and XIII), Y.J. Yuan's team at Tianjin University (led chromosomes V and X), Dai lab at Tsinghua University and Shenzhen Institute of Advanced Technology, CAS (led chromosome XII), Ellis lab at Imperial College London (led chromosome XI), Sakkie Pretorius's team at Macquarie University (led chromosomes XIV and XVI), Matthew Wook Chang's team at National University of Singapore (led chromosome XV), Bader and Boeke labs at Johns Hopkins University (led design and workflow), and Build-A-Genome undergraduate teams at Johns Hopkins University and Loyola University Maryland (contributed to chromosomes I, III, IV, VIII, and IX). The Sc2.0 consortium includes numerous other participants and are acknowledged on the project website www.syntheticyeast.org.

STAR★METHODS

Detailed methods are provided in the online version of this paper and include the following:

- KEY RESOURCES TABLE
- RESOURCE AVAILABILITY
  - Lead contact
  - Materials availability
  - Data and code availability
- EXPERIMENTAL MODEL AND SUBJECT DETAILS
- METHOD DETAILS
  - *SynVII* design and construction
  - Stress sensitivity assay
  - Omics analyses
  - Fitness defect mapping and reversion
  - Construction of aneuploid synVII strain
  - Genome stability analysis of aneuploid synVII strain
  - SCRaMbLE and screening of aneuploid synVII strain
  - Measurement of growth
  - Chromosome copy number determination by quantitative real-time PCR (qPCR)
  - Hi-C library construction and analysis
  - Gene expression profiling analysis of aneuploid SCRaMbLEd synVII strains
  - Flow cytometry analysis
- QUANTIFICATION AND STATISTICAL ANALYSIS

SUPPLEMENTAL INFORMATION

Supplemental information can be found online at https://doi.org/10.1016/j.xgen.2023.100364.

ACKNOWLEDGMENTS

This work is part of the international Synthetic Yeast Genome (Sc2.0) consortium. The project was supported by the National Key Research and Development Program of China (2018YFA0900100). This work was also supported by UK Biotechnology and Biological Sciences Research Council (BBSRC) grants BB/M005690/1, BB/P02114X/1 and BB/W014483/1; Engineering and Physical Sciences Research Council (EPSRC) EP/V05967X/1; and a Volkswagen Foundation "Life?" Initiative grant (ref. 94 771) (to Y.C.). J.D. was supported by a Royal Society Newton Advanced Fellowship (NAF\R2\180590) hosted by Y.C. This work was also supported by the National Natural Science Foundation of China (31800078 and 21901165); Science, Technology and Innovation Commission of Shenzhen Municipality (JCYJ20180507183534578); Guangdong Provincial Key Laboratory of Genome Read and Write (2017B030301011); Guangdong Provincial Academician Workstation of BGI Synthetic Genomics (2017B090904014); Tip-top Scientific and Technical Innovative Youth Talents of Guangdong Special Support Program (2019TQ05Y876), China; and Shenzhen Engineering Laboratory for Innovative Molecular Diagnostics (DRC-SZ [2016]884). We thank the DNA assembly automation platform of China National Genebank for the support on synthetic chunk assembly. L.M., J.S.B., and J.D.B. were supported by grants from the US National Science Foundation.

AUTHOR CONTRIBUTIONS

Conceptualization, Y.S. and Y.C.; funding and resources, Y.S. and Y.C.; data production, F.G., Yuerong Wang, Y.S., J. Zheng, Z.L., D.S., Y.D., W.D., T.L., R.S., H.Z., S.J., C.Z., S.C., T.C., and Yong Wang; data analyses, investigation, and visualization, Yun Wang, J. Zhang, J.G., Y.L., L.M., J.S.B., G.Z., X.S., and X.F.; writing – original draft, Y.S., X.F., Yun Wang, Yuerong Wang, J. Zheng, F.G., Y.C., and J.D.B.; writing – review & editing, all co-authors.

DECLARATION OF INTERESTS

J.D.B. is a founder and director of CDI Labs, Inc.; a founder of and consultant to Neochromosome, Inc.; and a founder and scientific advisory board member of and consultant to ReOpen Diagnostics, LLC. J.D.B. serves or served on the scientific advisory board of the following: Logomix, Inc., Sangamo, Inc., Modern Meadow, Inc., Rome Therapeutics, Inc., Sample6, Inc., Tessera Therapeutics, Inc., and the Wyss Institute. J.S.B. is a founder of Neochromsome, Inc.; is a consultant to Opentrons Labworks, Inc.; and serves on the scientific advisory board of Reflexion Pharmaceuticals, Inc.

INCLUSION AND DIVERSITY

We support inclusive, diverse, and equitable conduct of research.

Received: October 21, 2022
Revised: April 3, 2023
Accepted: July 6, 2023
Published: November 8, 2023

REFERENCES

1. Baker, D.J., Dawlaty, M.M., Wijshake, T., Jeganathan, K.B., Malureanu, L., van Ree, J.H., Crespo-Diaz, R., Reyes, S., Seaburg, L., Shapiro, V., et al. (2013). Increased expression of BubR1 protects against aneuploidy and cancer and extends healthy lifespan. Nat. Cell Biol. *15*, 96–102. https://doi.org/10.1038/ncb2643.

2. Ben-David, U., and Amon, A. (2020). Context is everything: aneuploidy in cancer. Nat. Rev. Genet. *21*, 44–62. https://doi.org/10.1038/s41576-019-0171-x.

3. Holland, A.J., and Cleveland, D.W. (2009). Boveri revisited: chromosomal instability, aneuploidy and tumorigenesis. Nat. Rev. Mol. Cell Biol. *10*, 478–487. https://doi.org/10.1038/nrm2718.

4. Nagaoka, S.I., Hassold, T.J., and Hunt, P.A. (2012). Human aneuploidy: mechanisms and new insights into an age-old problem. Nat. Rev. Genet. *13*, 493–504. https://doi.org/10.1038/nrg3245.

5. Pellman, D. (2007). Aneuploidy and cancer. Nature *446*, 38–39. https://doi.org/10.1038/446038a.

6. Bonney, M.E., Moriya, H., and Amon, A. (2015). Aneuploid proliferation defects in yeast are not driven by copy number changes of a few dosage-sensitive genes. Gene Dev. *29*, 898–903. https://doi.org/10.1101/gad.261743.115.

7. Rumble, B., Retallack, R., Hilbich, C., Simms Multhaup, G., Martins, R., Hockey, A., Montgomery, P., Beyreuther, K., and Masters, C.L. (1989). Amyloid A4 protein and its precursor in Down's syndrome and Alzheimer's disease. N. Engl. J. Med. *320*, 1446–1452.

8. Chen, Y., Chen, S., Li, K., Zhang, Y., Huang, X., Li, T., Wu, S., Wang, Y., Carey, L.B., and Qian, W. (2019). Overdosage of Balanced Protein Complexes Reduces Proliferation Rate in Aneuploid Cells. Cell Syst. *9*, 129–142.e5. https://doi.org/10.1016/j.cels.2019.06.007.

9. Oromendia, A.B., Dodgson, S.E., and Amon, A. (2012). Aneuploidy causes proteotoxic stress in yeast. Gene Dev. *26*, 2696–2708. https://doi.org/10.1101/gad.207407.112.

10. Terhorst, A., Sandikci, A., Keller, A., Whittaker, C.A., Dunham, M.J., and Amon, A. (2020). The environmental stress response causes ribosome loss in aneuploid yeast cells. Proc. Natl. Acad. Sci. USA *117*, 17031–17040. https://doi.org/10.1073/pnas.2005648117.

11. Torres, E.M., Williams, B.R., Tang, Y.-C., and Amon, A. (2010). Thoughts on Aneuploidy. Cold Spring Harbor Symp. Quant. Biol. *75*, 445–451. https://doi.org/10.1101/sqb.2010.75.025.

12. Tsai, H.-J., Nelliat, A.R., Choudhury, M.I., Kucharavy, A., Bradford, W.D., Cook, M.E., Kim, J., Mair, D.B., Sun, S.X., et al. (2019). Hypo-osmotic-like stress underlies general cellular defects of aneuploidy. Nature *570*, 117–121. https://doi.org/10.1038/s41586-019-1187-2.

13. Mulla, W., Zhu, J., and Li, R. (2014). Yeast: a simple model system to study complex phenomena of aneuploidy. FEMS Microbiol. Rev. *38*, 201–212. https://doi.org/10.1111/1574-6976.12048.

14. Beach, R.R., Ricci-Tam, C., Brennan, C.M., Moomau, C.A., Hsu, P.H., Hua, B., Silberman, R.E., Springer, M., and Amon, A. (2017). Aneuploidy Causes Non-genetic Individuality. Cell *169*, 229–242.e21. https://doi.org/10.1016/j.cell.2017.03.021.

15. Schindler, D., Dai, J., and Cai, Y. (2018). Synthetic genomics: a new venture to dissect genome fundamentals and engineer new functions. Curr. Opin. Chem. Biol. *46*, 56–62. https://doi.org/10.1016/j.cbpa.2018.04.002.

16. Mitchell, L.A., Wang, A., Stracquadanio, G., Kuang, Z., Wang, X., Yang, K., Richardson, S., Martin, J.A., Zhao, Y., Walker, R., et al. (2017). Synthesis, debugging, and effects of synthetic chromosome consolidation: synVI and beyond. Science *355*, eaaf4831. https://doi.org/10.1126/science.aaf4831.

17. Shen, Y., Wang, Y., Chen, T., Gao, F., Gong, J., Abramczyk, D., Walker, R., Zhao, H., Chen, S., Liu, W., et al. (2017). Deep functional analysis of synII, a 770-kilobase synthetic yeast chromosome. Science *355*, eaaf4791. https://doi.org/10.1126/science.aaf4791.

18. Wu, Y., Li, B.-Z., Zhao, M., Mitchell, L.A., Xie, Z.-X., Lin, Q.-H., Wang, X., Xiao, W.-H., Wang, Y., Zhou, X., et al. (2017). Bug mapping and fitness testing of chemically synthesized chromosome X. Science *355*, eaaf4706. https://doi.org/10.1126/science.aaf4706.

19. Xie, Z.-X., Li, B.-Z., Mitchell, L.A., Wu, Y., Qi, X., Jin, Z., Jia, B., Wang, X., Zeng, B.-X., Liu, H.-M., et al. (2017). Perfect" designer chromosome V and behavior of a ring derivative. Science *355*, eaaf4704. https://doi.org/10.1126/science.aaf4704.

20. Zhang, W., Zhao, G., Luo, Z., Lin, Y., Wang, L., Guo, Y., Wang, A., Jiang, S., Jiang, Q., Gong, J., et al. (2017). Engineering the ribosomal DNA in a megabase synthetic chromosome. Science *355*, eaaf3981. https://doi.org/10.1126/science.aaf3981.

21. Annaluru, N., Muller, H., Mitchell, L.A., Ramalingam, S., Stracquadanio, G., Richardson, S.M., Dymond, J.S., Kuang, Z., Scheifele, L.Z., Cooper, E.M., et al. (2014). Total Synthesis of a Functional Designer Eukaryotic Chromosome. Science *344*, 55–58. https://doi.org/10.1126/science.1249252.

22. Shen, Y., Stracquadanio, G., Wang, Y., Yang, K., Mitchell, L.A., Xue, Y., Cai, Y., Chen, T., Dymond, J.S., Kang, K., et al. (2016). SCRaMbLE generates designed combinatorial stochastic diversity in synthetic chromosomes. Genome Res. *26*, 36–49. https://doi.org/10.1101/gr.193433.115.

23. Blount, B.A., Gowers, G.-O.F., Ho, J.C.H., Ledesma-Amaro, R., Jovicevic, D., McKiernan, R.M., Xie, Z.X., Li, B.Z., Yuan, Y.J., and Ellis, T. (2018). Rapid host strain improvement by in vivo rearrangement of a synthetic yeast chromosome. Nat. Commun. *9*, 1932. https://doi.org/10.1038/s41467-018-03143-w.

24. Jia, B., Wu, Y., Li, B.-Z., Mitchell, L.A., Liu, H., Pan, S., Wang, J., Zhang, H.-R., Jia, N., Li, B., et al. (2018). Precise control of SCRaMbLE in synthetic haploid and diploid yeast. Nat. Commun. *9*, 1933. https://doi.org/10.1038/s41467-018-03084-4.

25. Luo, Z., Wang, L., Wang, Y., Zhang, W., Guo, Y., Shen, Y., Jiang, L., Wu, Q., Zhang, C., Cai, Y., and Dai, J. (2018). Identifying and characterizing SCRaMbLEd synthetic yeast using ReSCuES. Nat. Commun. *9*, 1930. https://doi.org/10.1038/s41467-017-00806-y.

26. Shen, M.J., Wu, Y., Yang, K., Li, Y., Xu, H., Zhang, H., Li, B.-Z., Li, X., Xiao, W.-H., Zhou, X., et al. (2018). Heterozygous diploid and interspecies SCRaMbLEing. Nat. Commun. *9*, 1934. https://doi.org/10.1038/s41467-018-04157-0.

27. Zhao, M., Zhao, Y., Yao, M., Iqbal, H., Hu, Q., Liu, H., Qiao, B., Li, C., Skovbjerg, C.A.S., Nielsen, J.C., et al. (2020). Pathway engineering in yeast for synthesizing the complex polyketide bikaverin. Nat. Commun. *11*, 6197. https://doi.org/10.1038/s41467-020-19984-3.

28. Liu, W., Luo, Z., Wang, Y., Pham, N.T., Tuck, L., Pérez-Pi, I., Liu, L., Shen, Y., French, C., et al. (2018). Rapid pathway prototyping and engineering using in vitro and in vivo synthetic genome SCRaMbLE-in methods. Nat. Commun. *9*, 1936. https://doi.org/10.1038/s41467-018-04254-0.

29. Tang, Y.-C., and Amon, A. (2013). Gene Copy-Number Alterations: A Cost-Benefit Analysis. Cell *152*, 394–405. https://doi.org/10.1016/j.cell.2012.11.043.

30. Torres, E.M., Dephoure, N., Panneerselvam, A., Tucker, C.M., Whittaker, C.A., Gygi, S.P., Dunham, M.J., and Amon, A. (2010). Identification of Aneuploidy-Tolerating Mutations. Cell *143*, 71–83. https://doi.org/10.1016/j.cell.2010.08.038.

31. Richardson, S.M., Mitchell, L.A., Stracquadanio, G., Yang, K., Dymond, J.S., DiCarlo, J.E., Lee, D., Huang, C.L.V., Chandrasegaran, S., Cai, Y., et al. (2017). Design of a synthetic yeast genome. Science *355*, 1040–1044. https://doi.org/10.1126/science.aaf4557.

32. Winzeler, E.A., Shoemaker, D.D., Astromoff, A., Liang, H., Anderson, K., Andre, B., Bangham, R., Benito, R., Boeke, J.D., Bussey, H., et al. (1999). Functional Characterization of the *S. cerevisiae* Genome by Gene Deletion and Parallel Analysis. Science *285*, 901–906. https://doi.org/10.1126/science.285.5429.901.

33. Oliveira, C.C., van den Heuvel, J.J., and McCarthy, J.E. (1993). Inhibition of translational initiation in Saccharomyces cerevisiae by secondary structure: the roles of the stability and position of stem-loops in the mRNA leader. Mol. Microbiol. *9*, 521–532. https://doi.org/10.1111/j.1365-2958.1993.tb01713.x.

34. Zhao, Y., Coelho, C., Hughes, A.L., Lazar-Stefanita, L., Yang, S., Brooks, A.N., Walker, R.S.K., Zhang, W., Lauer, S., Hernandez, C., et al. (2022).

Debugging and consolidating multiple synthetic chromosomes reveals combinatorial genetic interactions. Preprint at Biorxiv. https://doi.org/10.1101/2022.04.11.486913.

35. Hill, A., and Bloom, K. (1987). Genetic manipulation of centromere function. Mol. Cell Biol. *7*, 2397–2405. https://doi.org/10.1128/mcb.7.7.2397.

36. Potapova, T.A., Zhu, J., and Li, R. (2013). Aneuploidy and chromosomal instability: a vicious cycle driving cellular evolution and cancer genome chaos. Cancer Metastasis Rev. *32*, 377–389. https://doi.org/10.1007/s10555-013-9436-6.

37. Sheltzer, J.M., Blank, H.M., Pfau, S.J., Tange, Y., George, B.M., Humpton, T.J., Brito, I.L., Hiraoka, Y., Niwa, O., and Amon, A. (2011). Aneuploidy Drives Genomic Instability in Yeast. Science *333*, 1026–1030. https://doi.org/10.1126/science.1206412.

38. Zhang, H., Fu, X., Gong, X., Wang, Y., Zhang, H., Zhao, Y., and Shen, Y. (2022). Systematic dissection of key factors governing recombination outcomes by GCE-SCRaMbLE. Nat. Commun. *13*, 5836. https://doi.org/10.1038/s41467-022-33606-0.

39. Dymond, J.S., Richardson, S.M., Coombes, C.E., Babatz, T., Muller, H., Annaluru, N., Blake, W.J., Schwerzmann, J.W., Dai, J., Lindstrom, D.L., et al. (2011). Synthetic chromosome arms function in yeast and generate phenotypic diversity by design. Nature *477*, 471–476. https://doi.org/10.1038/nature10403.

40. Torres, E.M., Sokolsky, T., Tucker, C.M., Chan, L.Y., Boselli, M., Dunham, M.J., and Amon, A. (2007). Effects of Aneuploidy on Cellular Physiology and Cell Division in Haploid Yeast. Science *317*, 916–924. https://doi.org/10.1126/science.1142210.

41. Sheltzer, J.M., Torres, E.M., Dunham, M.J., and Amon, A. (2012). Transcriptional consequences of aneuploidy. Proc. Natl. Acad. Sci. USA *109*, 12644–12649. https://doi.org/10.1073/pnas.1209227109.

42. Pavelka, N., Rancati, G., Zhu, J., Bradford, W.D., Saraf, A., Florens, L., Sanderson, B.W., Hattem, G.L., and Li, R. (2010). Aneuploidy confers quantitative proteome changes and phenotypic variation in budding yeast. Nature *468*, 321–325. https://doi.org/10.1038/nature09529.

43. Harari, Y., Ram, Y., Rappoport, N., Hadany, L., and Kupiec, M. (2018). Spontaneous Changes in Ploidy Are Common in Yeast. Curr. Biol. *28*, 825–835.e4. https://doi.org/10.1016/j.cub.2018.01.062.

44. Chen, Y., Chen, Y., Shi, C., Huang, Z., Zhang, Y., Li, S., Li, Y., Ye, J., Yu, C., Li, Z., et al. (2018). SOAPnuke: A MapReduce Acceleration supported Software for integrated Quality Control and Preprocessing of High-Throughput Sequencing Data. GigaScience *7*, 1–6. https://doi.org/10.1093/gigascience/gix120.

45. Langmead, B., Trapnell, C., Pop, M., and Salzberg, S.L. (2009). Ultrafast and memory-efficient alignment of short DNA sequences to the human genome. Genome Biol. *10*, R25. https://doi.org/10.1186/gb-2009-10-3-r25.

46. McKenna, A., Hanna, M., Banks, E., Sivachenko, A., Cibulskis, K., Kernytsky, A., Garimella, K., Altshuler, D., Gabriel, S., Daly, M., and DePristo, M.A. (2010). The Genome Analysis Toolkit: A MapReduce framework for analyzing next-generation DNA sequencing data. Genome Res. *20*, 1297–1303. https://doi.org/10.1101/gr.107524.110.

47. Li, H., Handsaker, B., Wysoker, A., Fennell, T., Ruan, J., Homer, N., Marth, G., Abecasis, G., and Durbin, R.; 1000 Genome Project Data Processing Subgroup (2009). The Sequence Alignment/Map format and SAMtools. Bioinformatics *25*, 2078–2079. https://doi.org/10.1093/bioinformatics/btp352.

48. Milne, I., Stephen, G., Bayer, M., Cock, P.J.A., Pritchard, L., Cardle, L., Shaw, P.D., and Marshall, D. (2013). Using Tablet for visual exploration of second-generation sequencing data. Briefings Bioinf. *14*, 193–202. https://doi.org/10.1093/bib/bbs012.

49. Trapnell, C., Pachter, L., and Salzberg, S.L. (2009). TopHat: discovering splice junctions with RNA-Seq. Bioinformatics *25*, 1105–1111. https://doi.org/10.1093/bioinformatics/btp120.

50. Anders, S., and Huber, W. (2010). Differential expression analysis for sequence count data. Genome Biol. *11*, R106. https://doi.org/10.1186/gb-2010-11-10-r106.

51. Wen, B., Zhou, R., Feng, Q., Wang, Q., Wang, J., and Liu, S. (2014). IQuant: An automated pipeline for quantitative proteomics based upon isobaric tags. Proteomics *14*, 2280–2285. https://doi.org/10.1002/pmic.201300361.

52. Servant, N., Varoquaux, N., Lajoie, B.R., Viara, E., Chen, C.-J., Vert, J.-P., Heard, E., Dekker, J., and Barillot, E. (2015). HiC-Pro: an optimized and flexible pipeline for Hi-C data processing. Genome Biol. *16*, 259. https://doi.org/10.1186/s13059-015-0831-x.

53. Li, J., Zhang, W., and Li, X. (2018). 3D Genome Reconstruction with ShRec3D+ and Hi-C Data. IEEE ACM Trans. Comput. Biol. Bioinf *15*, 460–468. https://doi.org/10.1109/tcbb.2016.2535372.

54. Tong, A.H., Evangelista, M., Parsons, A.B., Xu, H., Bader, G.D., Pagé, N., Robinson, M., Raghibizadeh, S., Hogue, C.W., Bussey, H., et al. (2001). Systematic Genetic Analysis with Ordered Arrays of Yeast Deletion Mutants. Science *294*, 2364–2368. https://doi.org/10.1126/science.1065810.

55. Anders, K.R., Kudrna, J.R., Keller, K.E., Kinghorn, B., Miller, E.M., Pauw, D., Peck, A.T., Shellooe, C.E., and Strong, I.J.T. (2009). A strategy for constructing aneuploid yeast strains by transient nondisjunction of a target chromosome. BMC Genet. *10*, 36. https://doi.org/10.1186/1471-2156-10-36.

56. Warringer, J., and Blomberg, A. (2003). Automated screening in environmental arrays allows analysis of quantitative phenotypic profiles in Saccharomyces cerevisiae. Yeast *20*, 53–67. https://doi.org/10.1002/yea.931.

57. Dekker, J., Rippe, K., Dekker, M., and Kleckner, N. (2002). Capturing Chromosome Conformation. Science *295*, 1306–1311. https://doi.org/10.1126/science.1067799.

58. Mercy, G., Mozziconacci, J., Scolari, V.F., Yang, K., Zhao, G., Thierry, A., Luo, Y., Mitchell, L.A., Shen, M., Shen, Y., et al. (2017). 3D organization of synthetic and scrambled chromosomes. Science *355*, eaaf4597. https://doi.org/10.1126/science.aaf4597.

59. Brion, C., Pflieger, D., Souali-Crespo, S., Friedrich, A., and Schacherer, J. (2016). Differences in environmental stress response among yeasts is consistent with species-specific lifestyles. Mol. Biol. Cell *27*, 1694–1705. https://doi.org/10.1091/mbc.e15-12-0816.

60. Gasch, A.P., Spellman, P.T., Kao, C.M., Carmel-Harel, O., Eisen, M.B., Storz, G., Botstein, D., and Brown, P.O. (2000). Genomic Expression Programs in the Response of Yeast Cells to Environmental Changes. Mol. Biol. Cell *11*, 4241–4257. https://doi.org/10.1091/mbc.11.12.4241.

STAR★METHODS

KEY RESOURCES TABLE

REAGENT or RESOURCE	SOURCE	IDENTIFIER
Chemicals, peptides, and recombinant proteins		
Formulation SC Triple: SC, Triple Drop-Out –His, –Leu, –Ura	FORMEDIUM	DSCK1024
BD Difco™ Yeast Nitrogen Base without Amino Acids and Ammonium Sulfate	BD	233520
Lithium acetate	Sigma-Aldrich	517992-100G
D-(+)-Galactose	Sigma-Aldrich	G5388-100G
D-(+)-Glucose	Diamond	A100188-0005
Glycerol	Sigma-Aldrich	G5516-1L
Yeast Extract	Sangong Biotech	A515245-0500
Peptone	Sangong Biotech	A505247-0500
Agar	Sangong Biotech	A505255-0250
Ammonium sulfate	Diamond	A100191-0500
ssDNA	SIGMA	D1626-250MG
5-FOA	USBiological	F5050
Methyl methanesulfonate	Sigma-Aldrich	129925-5G
6-Azauracil	SIGMA	A1757-5G
Benomyl	Sigma-Aldrich	381586-5G
Camptothecin	SIGMA	C9911-250MG
H_2O_2	Sigma-Aldrich	88597-100ML-F
D-Sorbitol	BBI Life Sciences	A610491-0500
Cycloheximide	MCE	HY-12320/CS-4985
Hydroxyurea	SIGMA	H8627-25G
DTT	BBI Life Sciences	A620058-0025
Geldanamycin	AdooQ® Bioscience	A11025
Thiolutin	APExBIO	A4547
β-estradiol	Sigma-Aldrich	E8875-250MG
Lyticase	SIGMA	L2524-25KU
Zymolyase 100T	USBiological	37340-57-1
DpnII restriction enzyme	NEB	R0543S
T4 DNA ligase	NEB	M0202S
Igepal CA-630	Sigma-Aldrich	9002-93-1
Biotin-14-dATP	Invitrogen	19524016
XP beads	MGI	1000005279
Dynabeads MyOne Streptavidin T1 beads	Invitrogen	65601
NEB klenow exo minus	NEB	M0212S
Critical commercial assays		
E.A.N.A.Yeast RNA E.Z.N.A.®Yeast RNA Kit	OMEGA	R6870-02
Qubit™ dsDNA HS Assay Kit	INVITROGEN	Q32854
TB Green® Premix Ex Taq™ II (Tli RNase H Plus)	Clontech	RR820A
Maxima H Minus First Strand cDNA Synthesis Kit	Thermo Scientific	K1651
NucleoSpin Gel and PCR Clean-up	MACHEREY-NAGEL	740609.25
QIAprep Spin Miniprep Kit (250)	QIAGEN	27106

(*Continued on next page*)

CellPress
OPEN ACCESS

Continued

REAGENT or RESOURCE	SOURCE	IDENTIFIER
MGIEasyTM Universal DNA Library Prep Kit V1.0	MGI	1000006986
MGIEasy RNA Library Prep Set (96 RXN)	MGI	1000006384
iTRAQ reagent-8plex multiplex kit	Sigma-Aldrich	4381663
TMTpro^{TM} 16plex Label Reagent Set	Thermo Scientific	PIA44520
Experimental models: Organisms/strains		
S. cerevisiae: wild-type yeast strain BY4741/2	Jef Boeke laboratory	N/A
All other synthetic yeast strains used in this paper, listed Table S3	This paper	N/A
Deposited data		
Omics data of genome, transcriptome, and proteome	This paper	https://db.cngb.org/cnsa/; Accession number: CNP0002230
Hi-C data of synVII disome yeast	This paper	https://db.cngb.org/cnsa/; Accession number: CNP0002230
Sequence of design and physical *synVII*	This paper	https://db.cngb.org/cnsa/; Accession number: CNP0002230
Software and algorithms		
SOAPnuke v2.1.1	Chen et al.[44]	https://github.com/BGI-flexlab/SOAPnuke
Bowtie2 v2.2.5	Langmead et al.[45]	https://bowtie-bio.sourceforge.net/bowtie2/index.shtml
GATK v2.7	McKenna et al.[46]	https://github.com/broadinstitute/gatk/releases
SAMtools v0.1.19	Li et al.[47]	https://github.com/samtools/samtools
Tablet v1.21.08	Milne et al.[48]	https://ics.hutton.ac.uk/tablet/download-tablet/
TopHat v2.1.1	Trapnell et al.[49]	http://ccb.jhu.edu/software/tophat
DEseq2 v1.30.1	Anders et al.[50]	https://bioconductor.org/packages/release/bioc/html/DESeq2.html
Mascot 2.8.0	Matrix Science Ltd	https://www.matrixscience.com/
IQuant 2.0.1	Wen et al.[51]	https://sourceforge.net/projects/iquant/
HiC-Pro v3.1.0	Servant et al.[52]	https://github.com/nservant/HiC-Pro/tree/v3.1.0
ShRec3D+	Li et al.[53]	https://github.com/jbmorlot/ShRec-Exented
PyMoL v2.6.0a0	Schrödinger, LLC	https://pymol.org/2/
R v4.2.1	R Core Team	https://www.r-project.org/
Other		
Saccharomyces Genome Database (SGD)	SGD community	https://yeastgenome.org/

RESOURCE AVAILABILITY

Lead contact

Further information and request for reagents and resources should be directed to and will be fulfilled by the lead contact Yue Shen (shenyue@genomics.cn).

Materials availability

Requests for the generated plasmids and strains in this study should be directed to the lead contact Yue Shen (shenyue@genomics.cn).

Data and code availability

The data that support the findings of this study have been deposited into CNGB Sequence Archive (CNSA) of China National GeneBank DataBase (CNGBdb): CNP0002230.

EXPERIMENTAL MODEL AND SUBJECT DETAILS

Saccharomyces cerevisiae is used as the experimental model in the study. The haploid yeast strain BY4741 and BY4742 and diploid yeast strain BY4743 were used as wild type control.

METHOD DETAILS

SynVII design and construction

Methods of synthetic chromosome design, synthesis and assembly described previously were used in this study.[17,31] The final version of *synVII* is defined as yeast_chr07_3.57, with a total of ~11.89% sequence been modified. The sequence of synthetic chromosome *VII* was computationally segmented by BGI customized software "Segman" to 25~50 kb megachunks, then to 129~10 kb chunks and to final 485~3 kb minichunks for synthesis from scratch. More information on *synVII* design can be accessed on the synthetic yeast project website (www.syntheticyeast.org) and Table S1. A 2572 bp homologous region with I-*Sce*I site was designed on both semi-synthetic *synVII*s (*synVII-L* and *synVII-R*, strain ID: YSy088, YSy089) for integrating into the full-length *synVII* chromosome. The overlap region shared between adjacent chunks was designed to be ~800–1200 bp long. For megachunk integration, the ligation step was skipped and all 5–6 chunks (equal to 1 megachunk) were directly co-transformed into yeast to replace the corresponding wild-type sequence using homologous recombination followed by selection. Strains generated in this study are listed in Tables S2 and S3.

Stress sensitivity assay

Spot dilution assays were previously described.[17] Single colonies of BY4741, BY4742, and synVII (yeast_chr07_9.03, strain ID: YSy105) were cultured overnight in YPD medium at 30°C. Cells were 10-fold serial diluted and spotted onto various selective plates, with YPD medium plates at 30°C as control. All plates with drugs or adjusted pH (pH 4.0 and pH 9.0) were incubated at 30°C for 2–4 days. Plates were incubated at 25°C and 37°C for temperature stresses.

Omics analyses

Paired-end whole genome sequencing was performed on the synVII (yeast_chr07_9.03, strain ID: YSy105) with the BGIseq500 platform. A 200-400bp sequencing library was prepared according to BGI's DNA preparation protocol using the MGIEasyTM Universal DNA Library Prep Kit V1.0 (catalog number: 1000006986). The YSy105 and BY4741 strain both with 3 biological replicates were prepared using sample preparation methods established previously for transcriptome and proteome analysis.[17] For proteomics, proteins were labeled by iTRAQ reagent-8plex multiplex kit (catalog number: 4381663; BY4741 labeled in isobaric tag: 114, 115, 117; YSy105 labeled in isobaric tag: 118, 119, 121) and the peptides were fractionated with high pH RP method and analyzed by a Q Exactive HF-X mass spectrometer (Thermo Fisher Scientific, San Jose, CA) coupled with an online HPLC.

Sequencing reads QC, data processing and analysis were performed as described previously.[17] For genome sequencing analysis, after filtering low-quality reads with SOAPnuke,[44] clean reads were mapped to a reference sequence of the *synVII* yeast genome using Bowtie2 with default parameters.[45] Both GATK3.8[46] and SAMtools[47] pipelines were used to identify the variants, which were further validated by Tablet.[48] For transcriptomics, reads were mapped to genomes by TopHat,[49] and differentially expressed genes were analyzed by DEseq2.[50] For proteomics, Mascot and IQuant[51] were used for protein identification and quantification. Gene enrichment and coexpression enrichment analyses were performed using KEGG pathways and Gene Ontology annotations.

Fitness defect mapping and reversion

The well-established "synthetic genetic array" (SGA) analysis method, in which a query mutant is crossed with a pre-designed yeast gene-deletion mutant library[32,54] was applied for defect mapping in synVIIS intermediate strain (strain ID: YSy093). In Megachunk S, 20 of the 23 genes in Megachunk S have corresponding gene-deletions in the ~5000 yeast gene-deletion mutant SGA library (the remaining 3 are essential genes) and were recovered from the library. These mutant strains were mated with the synVIIS strain as well as a control synVIIT strain. By mating these deletion strains individually with the defective synVIIS strain, the resulting diploid strains with one gene copy deleted and another copy that cannot maintain proper function will pinpoint the cause of the observed growth defect namely *YGR159C*.

For the defect observed in megachunk W, YSy100 bearing full synthetic megachunk W was backcrossed to wild-type strain BY4742, followed by sporulation and tetrad dissection. The generated spores were then selected for further analysis by previously described method "PoPM"[18] and this helped to pinpoint the defect to synthetic variant sequences in *MTM1* and *RAD2* genes. Each of the designed sequence variants were individually introduced into the BY4741 strain to further examine their effects.

Construction of aneuploid synVII strain

The construction of aneuploid synVII strain was performed based on a previously described method involving transient nondisjunction of the target chromosome.[55] In this study, a conditional centromere construct (P_{GAL1}-*CEN7*) and a *ura3Δ3′-HIS3-ura3Δ5′* cassette were introduced on chromosome VII in the BY4742 strain (Figure 2A). The conditional centromere construct P_{GAL1}-*CEN7* was used to replace the *CEN7* sequence on chromosome VII for transiently blocking disjunction of chromosome VII in the presence

of galactose. In the *ura3Δ3′-HIS3-ura3Δ5′* cassette, a 390 bp direct repeat of a portion of *ura3* was designed in both *ura3Δ5′* and *ura3Δ3′* sequences. The galactose induction activates the *GAL1* promoter and blocks the function of *CEN7*, then the homologous recombination of the repeat of *ura3* can lead to excision and loss of *HIS3* and regeneration of functional *URA3*, which can lead to selection of aneuploid chromosome VII yeast cells YSy140 containing two copies of chromosome VII. *SynVII* was modified to carry the *LEU2* marker near the centromere, then through mating to YSy140 strain and sporulation, selection of His$^+$Leu$^+$ cells can lead to the final construction of aneuploid synVII. Whole genome sequencing of YSy142 revealed an extra copy of each chromosome I and III. By applying the same approach to generate YSy140, we successfully removed the extra copies of undesired chromosomes sequentially and leading to the auxotrophic marker on *chrVII* switched from *HIS3* to *MET15*. The chromosome sequence integrity of both *synVII* and chromosome VII was confirmed by whole genome sequencing on BGISEQ platform.

Genome stability analysis of aneuploid synVII strain

The aneuploid synVII strains (strain ID: YSy140 and YSy142) were streaked on SC–Leu–Ura and SC–Leu–Met plates respectively and incubated at 30°C for 5 days. Three single colonies of each strain were selected for successive subculture in 1mL SC medium for 210 generations at 30°C, followed by plating on the SC plates and then replica plating was performed on both SC–Leu–Ura and SC–Leu–Met plates. The loss of synthetic or wildtype chromosome VII was calculated by counting numbers of colonies present on the SC plate but not on corresponding selective plates.

SCRaMbLE and screening of aneuploid synVII strain

Aneuploid strains (strain ID: YSy142) with pSCW11-*CreEBD-URA3* plasmid were cultured in SC–Ura–Leu–Met liquid medium overnight at 30°C. Cells were inoculated into SC–Ura–Leu–Met liquid medium with 1 μM β-estradiol and cultured for 24 h at 30°C. Then SCRaMbLEd cells were plated on SC–Leu–Met+0.01 μg/mL cycloheximide plates followed by 4 days incubation at 30°C, with unSCRaMbLEd cells exposed in the same condition as phenotypic control. Single colonies with recovered phenotype compared to unSCRaMbLEd cells were selected for further phenotype tests and functional analyses.

Measurement of growth

Single colony of each SCRaMbLEd strains was grown in SC–Leu–Met liquid medium in 2.2 mL 96-deep well plate at 30°C for overnight. ~300 colonies for each strain were plated onto YPD and YPD+0.01 μg/mL cycloheximide agar plates respectively followed by incubating at 30°C for 2 days. Then, the colonies on the plates were automated identified, photographed, and processed using software that measures colony diameter in terms of millimeters by the Precision Colony Picker (model: PIXL, Singer Instruments). The relative colony size of each SCRaMbLEd strain was determined by the colony size (diameter) of individual colonies respectively divided the mean of colony size of parental disomic strain YSy142. The growth recovery rate was defined as the recovery degree of the colony size by comparison to its parental strain (reference). It was calculated by comparing average colony size (from ≥200 single colonies) of each strain to that of the unSCRaMbLEd aneuploid yeast parent YSy142. The formula is as follows:

$$\text{Growth recovery rate}(\%) = \frac{Si - Sr}{Sr} \times 100\%$$

Si = Colony size of strain i;

Sr = Colony size of corresponding parental strain r;

The growth curve was measured using a Microbiology Reader Bioscreen C system (Oy Growth Curve Ab Ltd). Overnight culture was inoculated into 300 μL of liquid medium (~0.01 OD). The medium was added as blank control. The optical density (OD) was automatically measured every 20 min at 30°C for 48 h following the manual of protocol. The doubling time of each yeast strains was estimated in the method described previously.[56] The OD value was transformed by log10. Then the largest slope (k) was calculated using linear regression method in R (version 3.6.3) by every 7 consecutive measurements for the whole growth curve. The doubling time (DT) was calculated according to the formula: DT = log10 (2)/k.

Chromosome copy number determination by quantitative real-time PCR (qPCR)

To determine the copy number of *chrI* and *chrIII*, an essential gene *YBR136W* on *chrII* was chosen as internal control, and *YAR007C*, *YCR052W* as targets on *chrI*, *chrIII* respectively. qPCR was conducted with 10ng genomic DNA in duplicate in a 20 μL reaction using the TB Green Premix Ex Taq II (Tli RNase H Plus, Takara) and the StepOnePlus Real-Time PCR System (ABI). Primer sequences: *YBR136W* forward primer 5′-TGGAACGTATTGGGGCTGAC-3′, reverse primer 5′- AGTCAGCGTCTG CTTGTTCA-3'; *YAR007C* forward primer 5′-GTGTGACGGATTTTGGTGGC-3′, reverse primer 5′-TGATGAAGTTTGCGTTGCGG-3'; *YCR052W* forward primer 5′-TTCCAACGATGCCGAAGACA-3′, reverse primer 5′-ACTTCACCCAATTCGG GCTT-3'.

Hi-C library construction and analysis

Hi-C libraries were generated using a protocol adapted from previous studies.[57,58] 1 × 10^9 cells were collected and washed from overnight cell culture, cells were cross-linked with 6 mL 3% formaldehyde in 1xPBS (137 mM NaCl, 2.7 mM KCl, 10 mM Na_2HPO_4, 1.8 mM KH_2PO_4) for 30 min, and quenched by adding 0.66 mL 2.5 M glycine (working concentration is 250 mM) at room temperature for 5 min and then keep on ice for 15 min. Cells were washed with 10 mL 1xPBS for 3 times and, pelleted cells

were dissolved in 10 mL 1 M sorbitol with 5 mM DTT and 1 mg/mL Zymolyase 100T and incubated at 30°C for 30-60 min to remove cell wall. Cells were lysed with 3 mL 0.2% Igepal CA-630 and 10 mM Tris-HCl, followed by 500 μL 0.5% SDS treatment at 62°C for 10 min and 250 μL 10% Triton X-100 treatment at 37°C for 15 min, then cells were washed and resuspended with restriction buffer (NEB3.1). Cross-linked DNA was digested at 37°C overnight with 200 units of DpnII restriction enzyme (NEB) in a 500 μL reaction. The digestion mix was placed at 62°C for 20 min, and put on ice immediately. DNA ends were repaired with 25 μL 0.4 mM biotin-14-dATP at 23°C for 4 h, and ligated with 1000 U NEB T4 ligase at 16°C for 6 h. DNA decrosslinking was performed through an overnight incubation at 65°C with 25 μL 20 mg/mL proteinase K added to a total reaction volume of 1.2 mL. DNA was purified on XP beads, and unligated biotin ends were removed using T4 DNA polymerase. DNA was sheared and Biotin-tagged DNA were pull-down with Dynabeads MyOne Streptavidin T1 beads, repaired ends with T4 DNA polymerase and tagging a tail with NEB klenow exo minus, filled in adaptors with barcode oligo mix, PCR amplified resulting DNA and collected DNA with 300–600 bp size fragments, denatured DNA at 95°C for 3 min and ssDNA cyclization with splint Oligo and T4 DNA ligase. The resulting Hi-C libraries were processed into whole genome sequencing on BGISEQ platform. The Hi-C data's raw and normalized contact count matrices were calculated at a resolution of 10 kb by HiC-Pro v3.1.0 using default parameters.[52] The 3D genome architecture was constructed by ShRec3D+[53] using the normalized chromosomal contacts, and visualized them by PyMoL v2.6.0a0 (Schrödinger, LLC.).

Gene expression profiling analysis of aneuploid SCRaMbLEd synVII strains

The genome reconstruction of aneuploid SCRaMbLEd synVII was performed using the previously described method.[22] The reference sequence of YSy142 was updated by adding the *synVII* sequence to the other chromosomes of the BY4741 reference genome.

For the transcriptome analysis, the environmental stress response (ESR) gene sets identified in previous studies[59,60] were used for expression level analysis. The significance of GO terms in differentially expressed ESR genes was individually identified using the hyper-geometric test with false discovery rate (FDR) correction and the threshold p value < 0.001.

Flow cytometry analysis

Asynchronous log-phase cells were fixed with 70% ethanol for 1 h at room temperature. Then cell pellets were resuspended in 50 mM sodium citrate (pH 7.0). Samples were briefly sonicated and placed on ice, followed by Rnase A (0.25 mg/mL) treatment for 1 h at 50°C. Cells were washed with 50 mM sodium citrate (pH 7.0) and resuspended into the same solution. Propidium iodide (16 μg/mL) was added to the cells and incubated at room temperature for 30 min. Samples were analyzed with BD FACSCelesta Cell Analyzer.

QUANTIFICATION AND STATISTICAL ANALYSIS

Various statistical tests were employed to calculate p values, as indicated in the STAR Methods section, figure legend, or text, where appropriate. In general, results were considered statistically significant when $p < 0.001$ or FDR <0.01, unless stated otherwise. Specifically, in transcriptome level, the genes were assessed for statistical significance if FDR <0.01 and p fell below the threshold of the 5% Family Wise Error Rate (FWER) after Bonferroni correction (threshold = 7.62E-6). Statistical analyses were conducted using R (v4.2.1).

Cell Genomics, Volume *3*

Supplemental information

Dissecting aneuploidy phenotypes by constructing Sc2.0 chromosome VII and SCRaMbLEing synthetic disomic yeast

Yue Shen, Feng Gao, Yun Wang, Yuerong Wang, Ju Zheng, Jianhui Gong, Jintao Zhang, Zhouqing Luo, Daniel Schindler, Yang Deng, Weichao Ding, Tao Lin, Reem Swidah, Hongcui Zhao, Shuangying Jiang, Cheng Zeng, Shihong Chen, Tai Chen, Yong Wang, Yisha Luo, Leslie Mitchell, Joel S. Bader, Guojie Zhang, Xia Shen, Jian Wang, Xian Fu, Junbiao Dai, Jef D. Boeke, Huanming Yang, Xun Xu, and Yizhi Cai

Supplemental information

Dissecting aneuploidy phenotypes by constructing Sc2.0 chromosome VII and SCRaMbLEing synthetic disomic yeast

Yue Shen, Feng Gao, Yun Wang, Yuerong Wang, Ju Zheng, Jianhui Gong, Jintao Zhang, Zhouqing Luo, Daniel Schindler, Yang Deng, Weichao Ding, Tao Lin, Reem Swidah, Hongcui Zhao, Shuangying Jiang, Cheng Zeng, Shihong Chen, Tai Chen, Yong Wang, Yisha Luo, Leslie Mitchell, Joel S. Bader, GuojieZhang, Xia Shen, Jian Wang, Xian Fu, Junbiao Dai, Jef D. Boeke, Huanming Yang, Xun Xu, and Yizhi Cai

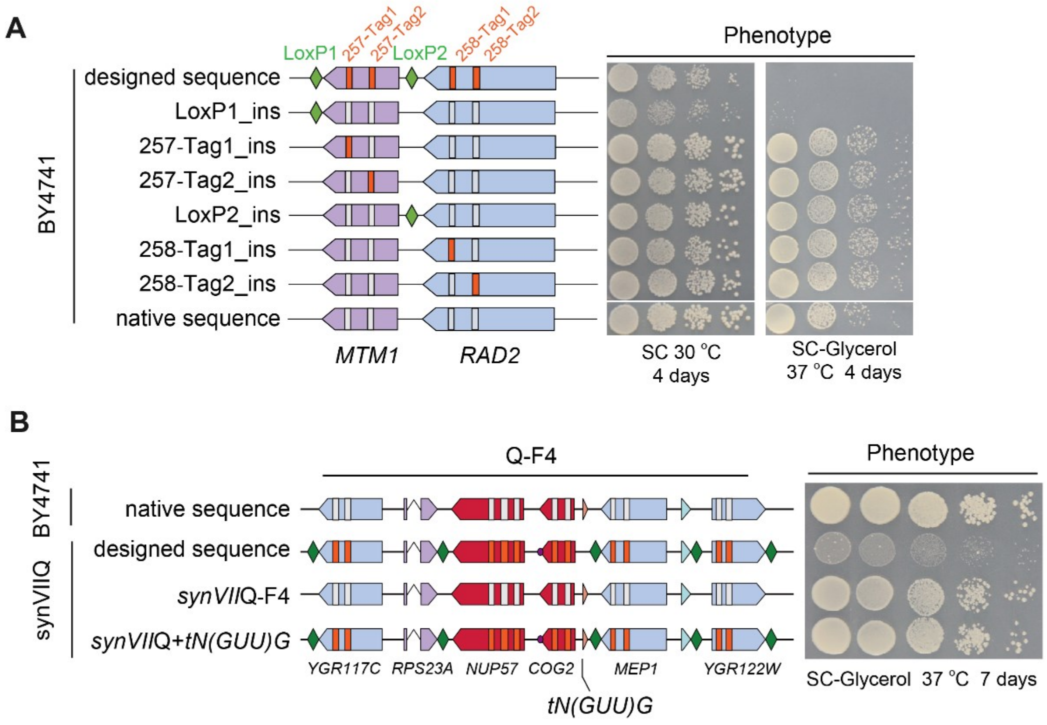

Figure S1. Debugging of W and Q regions in *synVII*, related to Figure 1. (A) Systematic dissection of defect origin in megachunk W by introducing each individual design feature into the BY4741 strain for phenotypic assay showing the loxPsym site at the 3'-UTR of *MTM1* causes the defect. (B) Spotting assay with ten-fold serial dilutions of BY4741, synVIIQ intermediate strain andits derived strains with chunk 4 and tRNA gene *tN(GUU)G* in chunk 4 replaced by native sequence showing the addition of tRNA gene *tN(GUU)G* recovered the phenotype. The arrows represent gene order and orientations (red indicates essential genes, purple is fast-growth and blue represents non-essential). Green diamonds represent loxPsym sites embedded downstream of the stop codons. Vertical orange and white bars represent synthetic and wild-type PCRTags respectively.

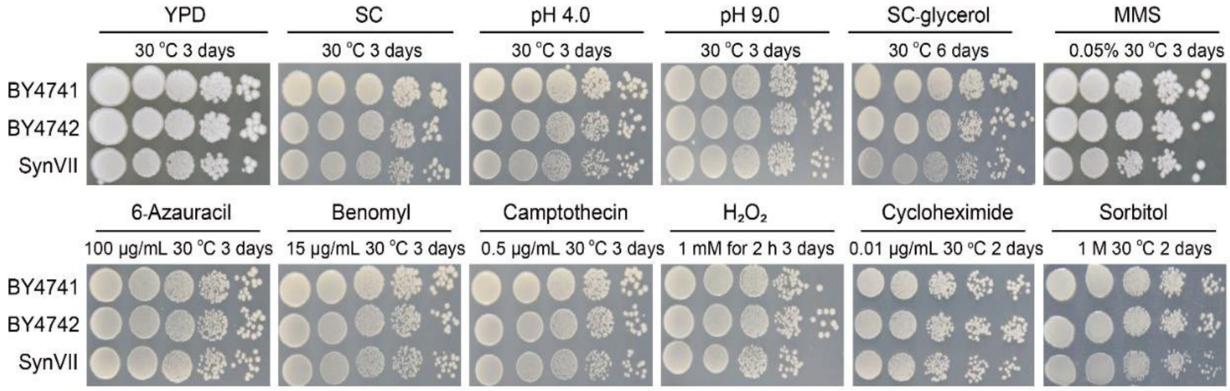

Figure S2. Phenotypic assays of synVII on different media in comparison with wild-type BY4741 and BY4742, related to Figure 1F and 1G. 10-fold serial dilutions of overnight cultures of selected strains were used for plating. From left to right: YPD at 30 °C; SC at 30 °C; low pH YPD (pH 4.0); high pH YPD (pH 9.0); SC-Glycerol; YPD + MMS; SC + 6-azauracil; SC + benomyl; SC + camptothecin; SC + H_2O_2 (1 mM, 2 hours pretreatment); SC + cycloheximide; SC + sorbitol; (YPD, yeast extract peptone dextrose; SC, synthetic complete; MMS, methyl methanesulfonate).

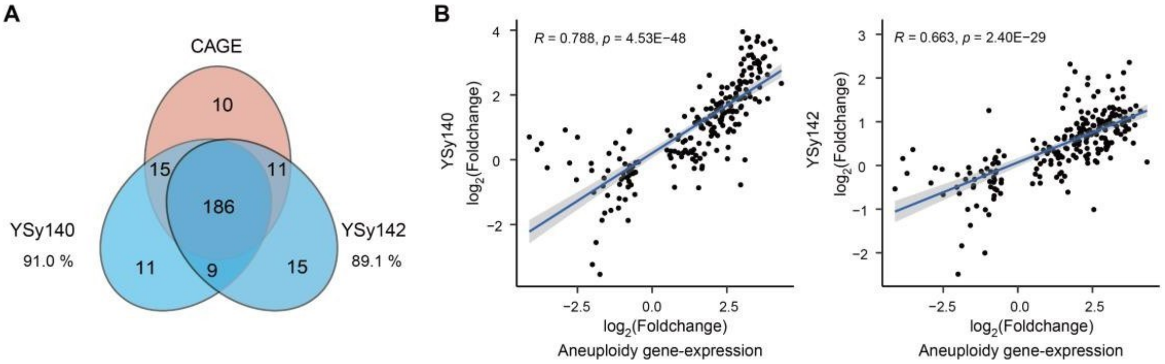

Figure S3. Comparison of the aneuploidy gene-expression in CAGE, related to Figure 2C. (A) Venn diagrams showing common genes with consistent differential gene expression among YSy140, YSy142 and aneuploidy gene-expression in CAGE. The percentage represents the consistency rate. The differential gene expression of the aneuploidy gene-expression, CAGE genes (n=222) originate from a previous study[10]. CAGE, common aneuploidy gene-expression. (**B**) Correlation of gene differential expression in YSy140 and YSy142 compared with aneuploidy gene-expression in CAGE genes. R indicates the Pearson's correlation. Solid blue line: fitted curve (ggplot:geom_point), geom_smooth (method=lm, se=TRUE). Gray area: 95% confidence range for fitted curve.

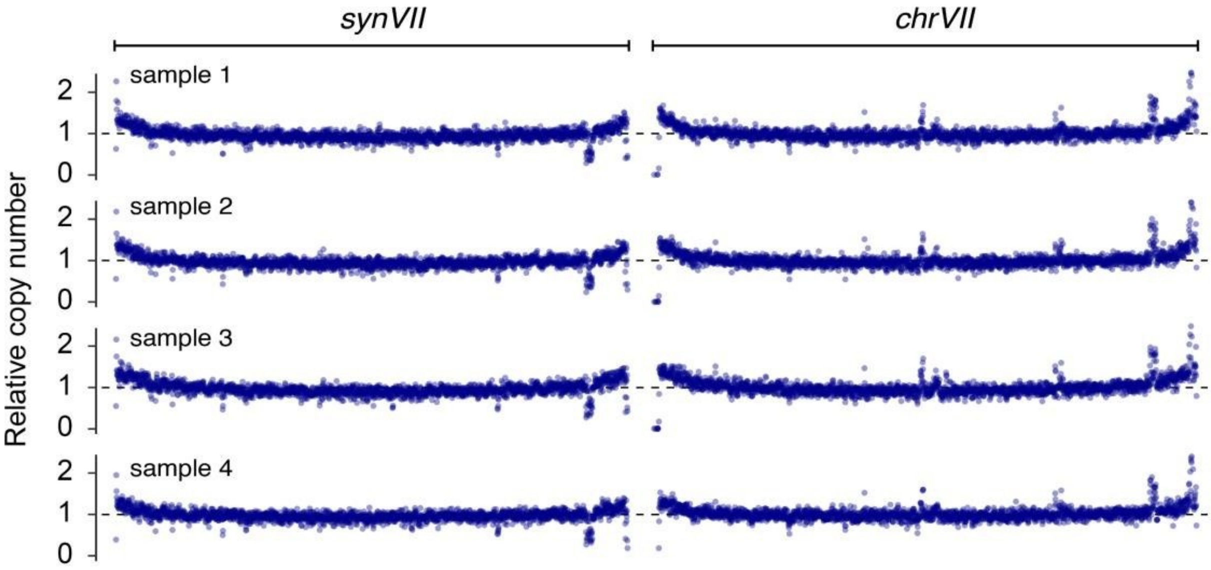

Figure S4. Verification of sequence integrity in disomic strain YSy142, related to Figure 2D. The relative sequencing depth of YSy142 colonies after batch transfer for 220 generations. The relative sequencing depth was calculated in 500-bp windows by comparing to the average of sequencing depth of the whole genome.

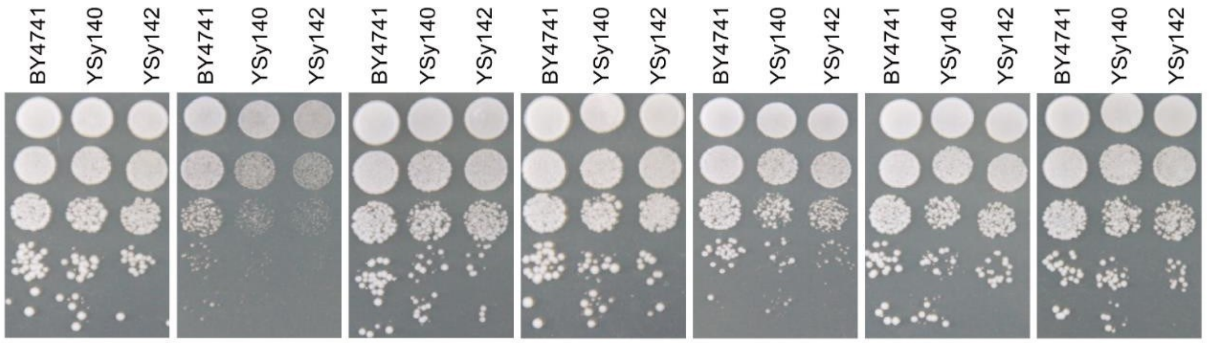

Figure S5. Spotting assay with ten-fold serial dilutions of disomic yeasts YSy140 and YSy142 compared to euploid strain BY4741 under selected conditions, related to Figure 3A. From leftto right: YPD at 30 °C; YPD + hydroxyurea (HU, 100 mM) at 30 °C; YPD + cycloheximide (Cyc, 0.01 μg/mL) at 30 °C; YPD + DL-Dithiothreitol (DDT, 2.5 mM pretreating 1 h); YPD + methyl methanesulfonate (MMS, 0.01% v/v) at 30 °C; YPD + geldanamycin (Geld, 1 μM) at 30 °C; YPD + thiolutin (Thio, 0.5 μg/mL) at 30 °C; (YPD, yeast extract peptone dextrose).

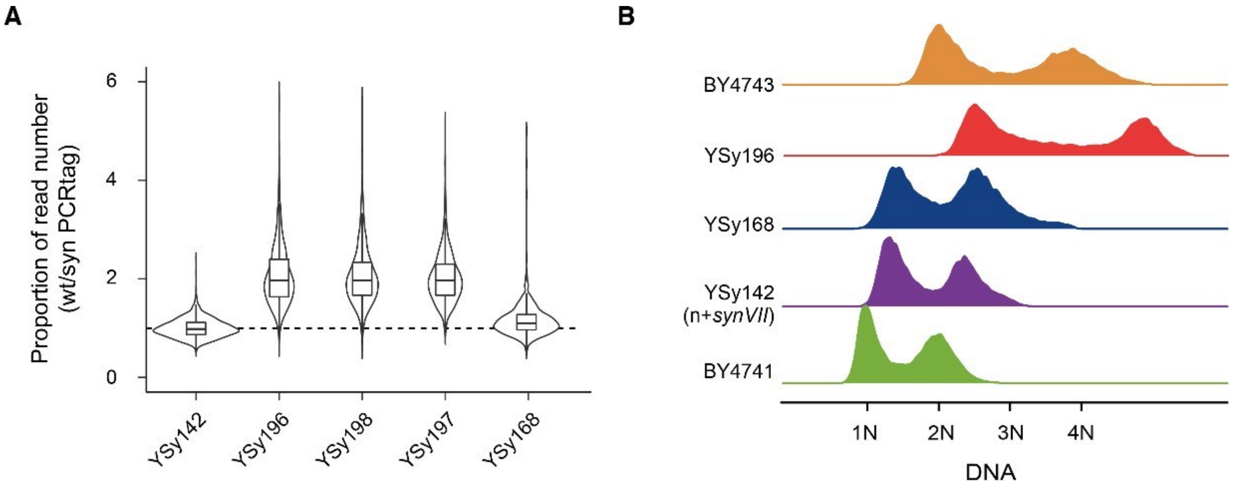

Figure S6. Karyotype analysis of SCRaMbLEd yeasts by whole genome sequencing analysis and flow cytometry, related to Figure 3. (A) The boxplot and violin plot present the proportion of reads for each wild-type (wt) versus synthetic (syn) PCRTags. The proportion is normalized by the mean of YSy142. The PCRtag sites of deleted region by SCRaMbLE is not included in the analysis. (B) For flow cytometry, asynchronous log-phase cells were analyzed (~15000 to 18000 cells/ each sample).

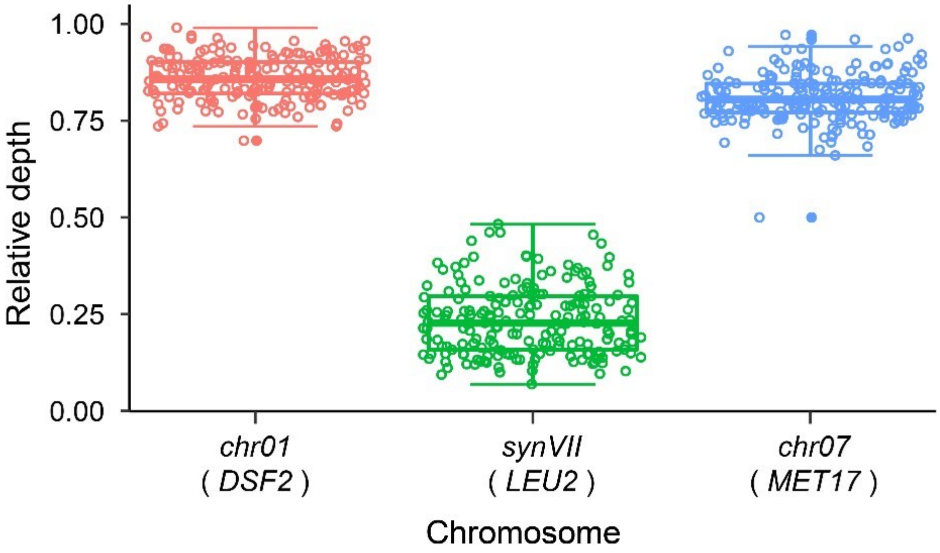

Figure S7. Relative average sequencing depth of 195 SCRaMbLEd strains with circular *synVII*, related to Figure 3. Deep sequencing coverage analysis revealed the lower depth of circular*synVII* in comparison with *chrVII* and *chrI* (control). The relative depths were quantified by *DSF2* gene on *chrI*, *LEU2* gene on *synVII* and *MET17* geneon *chrVII* respectively. Each dot represents one SCRaMbLEd strain.

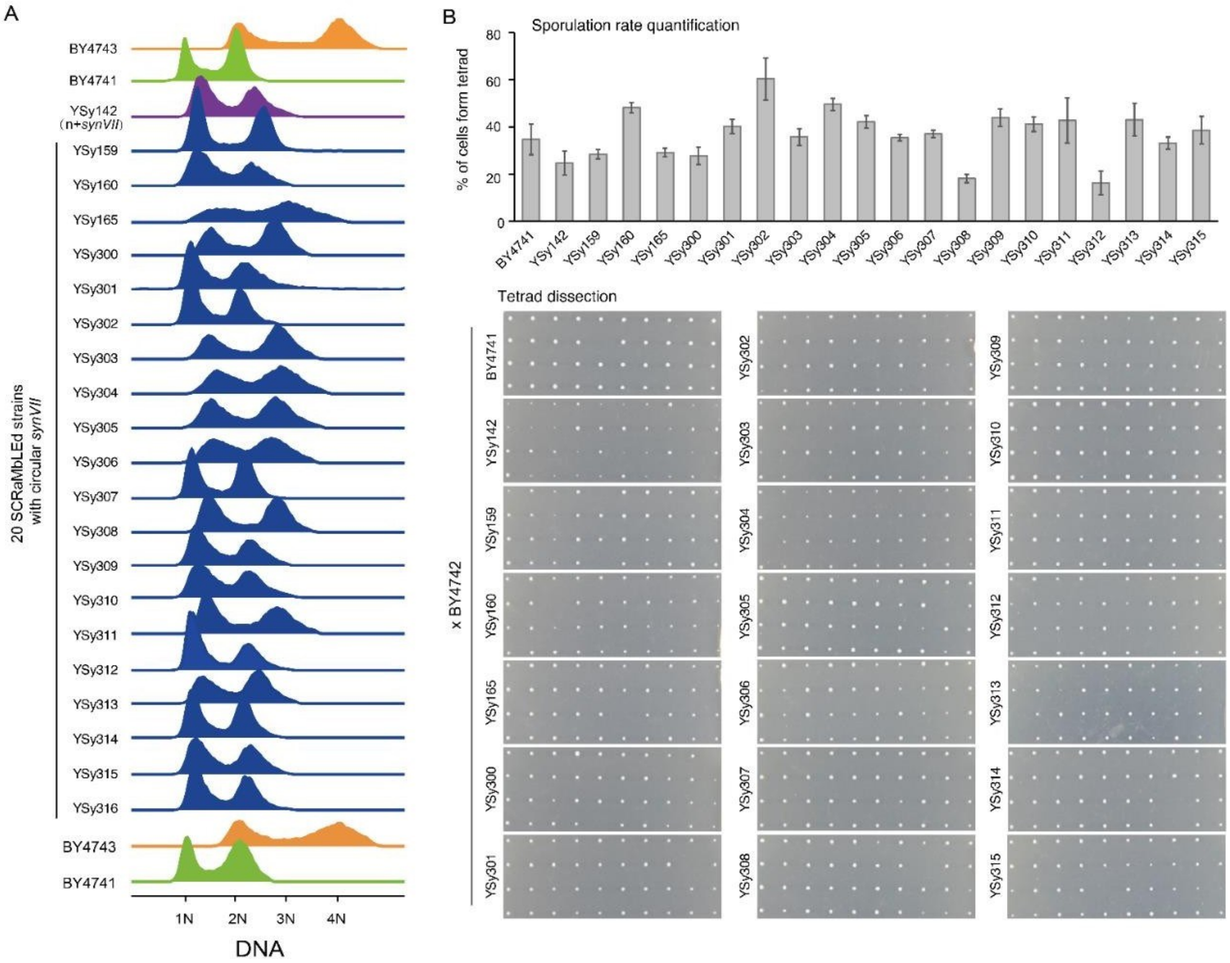

Figure S8. Karyotype validation by flow cytometry and sporulation in SCRaMbLEd strains with circular *synVII* chromosome, related to Figure 3. (A) For flow cytometry, ~15000 to 18000 cells were sorted. (B) For sporulation all selected samples were first mated to BY4742, then followed by sporulation and tetrad dissection (for each sample, 300 cells were counted at least for sporulation rate quantification, 10 tetrads were selected for dissection).

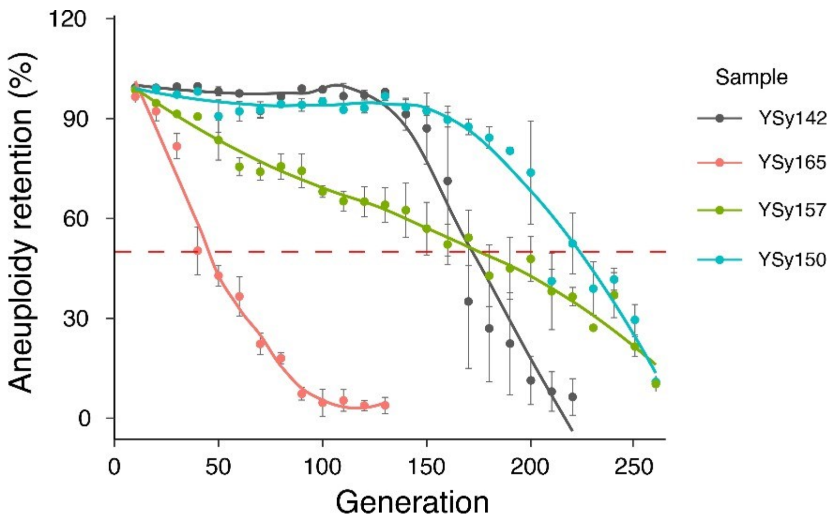

Figure S9. Genome stability analysis of SCRaMbLEd strains with circular *synVII* chromosome, related to Figure 3. Three representative strains with distinct *synVII* chromosome content and size were selected for genome stability assay by batch transfer for ~230 generations. YSy142 (n + *synVII*) is used as control.

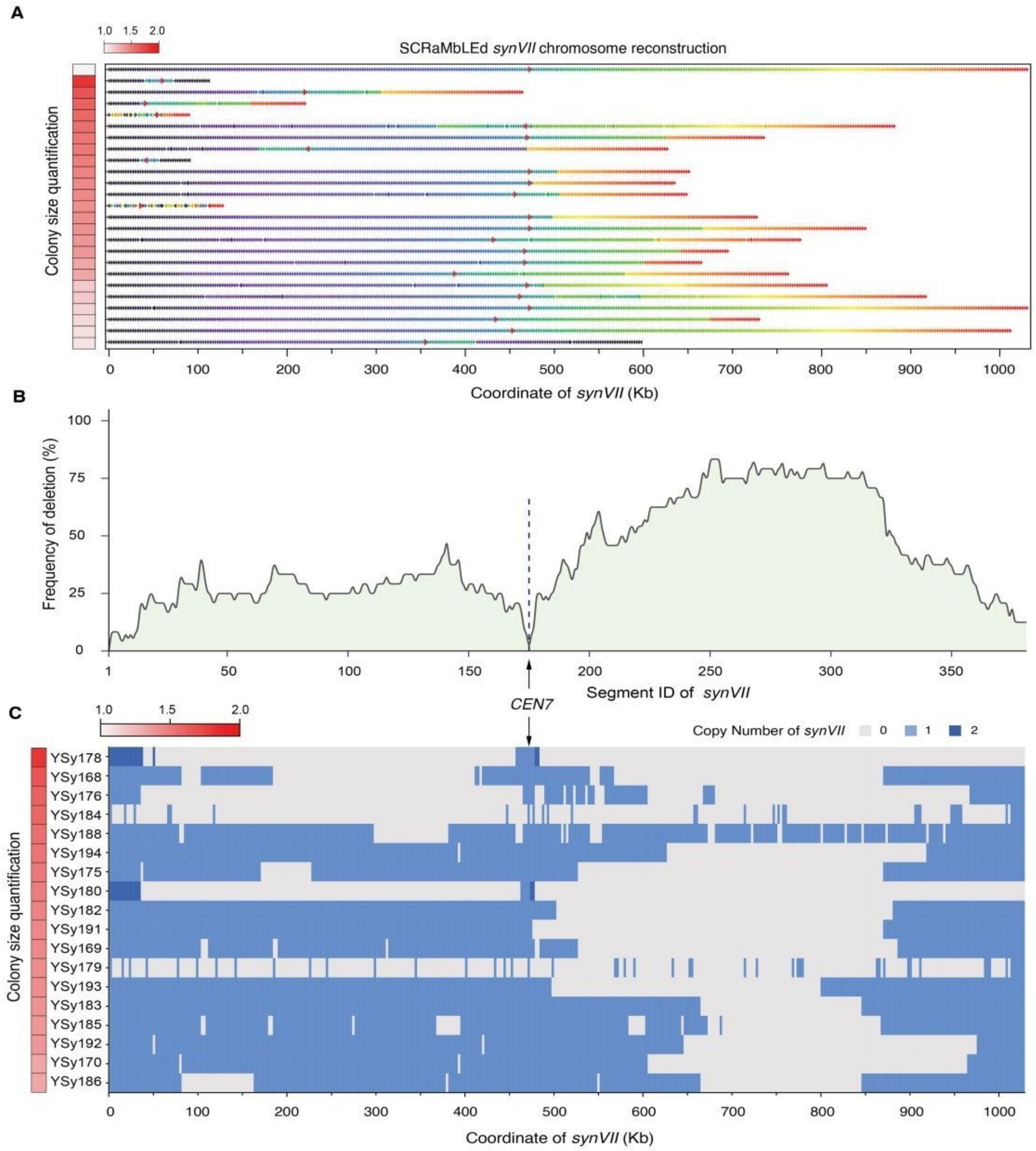

Figure S10. Deletion distribution across entire synthetic chromosome VII, related to Figure 3 and 5B. (**A**) Rearrangements were observed in the 24 SCRaMbLEd aneuploid yeasts with linear *synVII*. Each SCRaMbLE strain is represented as a sequence of arrows (SCRaMbLE-gram). The color and direction of each arrow indicates the segment number in the parental chromosome and its orientation. A red border denotes a segment containing *CEN7*. Y-axis shows the relative average phenotypic recovery rate of each SCRaMbLEd strain in comparison to that of YSy142 represented by color scale ($n \geq 200$). (**B**) Deletion hotspot observed in the right arm region of *synVII*. Y-axis represents the percentage of SCRaMbLEd strains that with corresponding segment been deleted. (**C**) The fate of each segment flanked by two loxPsym sites in each strain is indicated as deleted (gray) and one copy with no change (light blue).

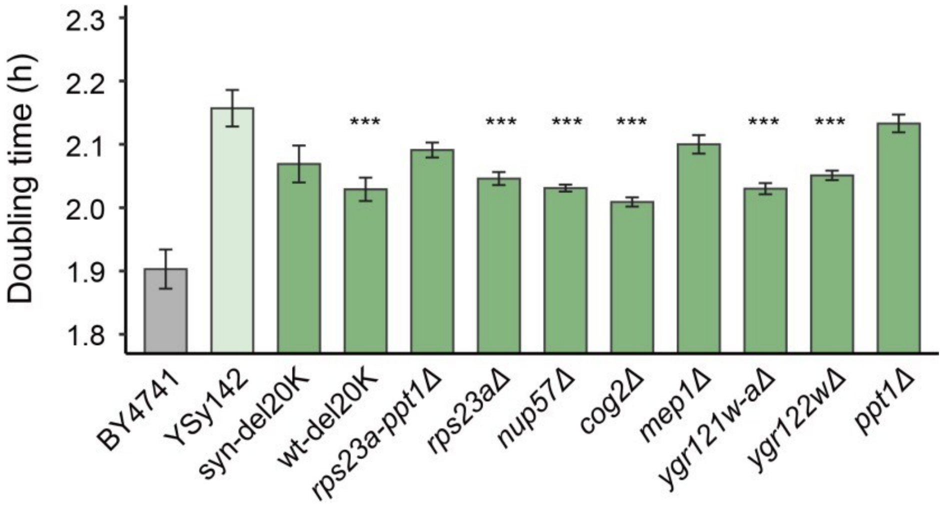

Figure S11. Doubling time measurement of reconstructed strains carrying the removal of regions/genes under normal condition, related to Figure 5C. Doubling time measurement of YSy142, *syn-del20K*, *wt-del20K*, and YSy142 carrying *syn-del12K* as well as the single gene knock-out in YPD medium at 30 °C. The error bar indicates standard error of the mean (SEM) in seven biological replicates. The P-value was calculated using single-tailed student t-test. *** P-value < 0.01.

Table S1. Summary of *synVII* design, related to STAR Methods.

Modification	Design feature	Number	Base alteration
Replacement	wild type telomere -> Universal Telomere Cap[a]	2	21094 -> 1378
	705 pairs of WT PCR tags and 705 pairs of SYN PCRTags	1410	17876
	stop codon TAG -> TAA	126	126
	"landmark" restriction sites (removal or introduction)[b]	261	596
	repeatsmash of gene	3	2147
Deletion	transposable element region[c]	20	51111
	gene	14	12232
	tRNA	36	2887
	intron[d]	23	3062
Insertion	LoxPsym site	380	12920

a. Wild-type telomeres together with its adjacent subtelomeric regions are replaced with universal telomere caps.

b. Unique restriction sites were generated in synVII either by introduction of synonymous mutations in ORFs, or by removal of redundant sites by the same method to leave only one pre- existing instance of a given site in the synVII sequence.

c. Transposable element region include these functional features of LTR retrotransposons, transposable element genes and long terminal repeats.

d. Eleven introns were retained as these introns reside in ribosomal subunit coding genes.

Table S2. Chromosome version used in this paper, related to STAR Methods.

Version name	Strain number	Comment	Details
yeast_chr07_3_56	NA	Original design sequence	Final design by BioStudio
yeast_chr07_3_57	NA	Updated design sequence	Swapped two genes of TAG to TAA (*YGL226C-A* and *YGL087C*)
yeast_chr07_9_01	YSy100	synVII draft strain, with 1 TAG stop codons, 7 loxPsym sites missing (one site is the consequence of debugging, 5 sites locate wild-type region), 0 wild-type PCRTags, 42 point mutations causing amino acid changes, 4505 bp wild-type region from *YGR290W* to right telomere;	Missing loxPsym sites: 409978-410011, 768480-768513, 953939-953972, 955439-955472.<br>Remaining TAG stop codons: 639475 A->G<br>Point mutations that cause amino acid changes: 35647 G->A, 63955 T->C, 118823 G->A, 211372 A->T, 228617 G->A, 228865 G->A, 228978 A->G, 280790 A->T, 290392 C->T, 294107 G->A, 302039 C->T, 319501 G->A, 319502 G->A, 394086 T->C, 452503 A->G, 513232 A->G, 576521 T->A, 580950 A->C, 581229 A->G, 582191 T->C, 586977 A->T, 591572 T->C, 601536 T->C, 602470 G->A, 638403 T->A, 638759 A->G, 640573 A->G, 640791 A->G, 641509 A->C, 642293 T->G, 662375 A->T, 666764 T->C, 671909 ATT->A, 692026 C->A, 693273 A->G, 694871 T->C, 697270 T->C, 697348 A->G, 747229 C->T, 773455 C->G, 787845 G->T, 992161 A->G<br><br>Wild-type region between 1024448 and 1028925
yeast_chr07_9_02	YSy101	synVII draft strain, with 1 TAG stop codons, 11 loxPsym sites missing (one site is the consequence of debugging, nine sites locate wild-type region), 18 wild-type PCRTags (locate wild-type region), 42 point mutations causing amino acid changes, 12563 bp wild-type region from *YGR252W* to	Missing loxPsym sites: 409978-410011, 768480-768513, 945933-945966, 951991-952024, 953939-953972, 955439-955472, 1024448-1024481, 1024833-1024866, 1026944-1026977, 1028314-1028347, 1028616-1028649.<br>Remaining TAG stop codons: 639475 A->G<br>Wild-type PCRTags: 945146-945173 YGR252W_1_synF, 945590-945617 YGR252W_1_synR, 946550-946577 YGR253C_1_synF, 946742-946769 YGR253C_1_synR, 948986-949013 YGR254W_1_synF, 949238-949265 YGR254W_1_synR, 950922-950949 YGR255C_1_synF, 951183-951210 YGR255C_1_synR, 952735-952756 YGR256W_1_synF, 952927-952954 YGR256W_1_synR, 954240-954267 YGR257C_1_synF, 954705-954732 YGR257C_1_synR, 955686-955713 YGR258C_1_synF, 955950-955977 YGR258C_1_synR, 956349-956376 YGR258C_2_synF, 956796-956823 YGR258C_2_synR, 957492-957519 YGR258C_3_synF, 957681-957708 YGR258C_3_synR<br><br>Point mutations that cause amino acid changes: 35647 G->A, 63955 T->C, 118823 G->A, 211372 A->T, 228617 G->A, 228865 G->A, 228978 A->G,

		YGR258C, 4505 bp wild-type region from *YGR290W* to right telomere	280790 A->T, 290392 C->T, 294107 G->A, 302039 C->T, 319501 G->A, 319502 G->A, 394086 T->C, 452503 A->G, 513232 A->G, 576521 T->A, 580950 A->C, 581229 A->G, 582191 T->C, 586977 A->T, 591572 T->C, 601536 T->C, 602470 G->A, 638403 T->A, 638759 A->G, 640573 A->G, 640791 A->G, 641509 A->C, 642293 T->G, 662375 A->T, 666764 T->C, 671909 ATT->A, 692026 C->A, 693273 A->G, 694871 T->C, 697270 T->C, 697348 A->G, 747229 C->T, 773455 C->G, 787845 G->T, 992161 A->G<br><br>Wild-type region: 945146-957708 ,1024448-1028925
yeast_chr07_9_03	YSy105	Repaired 4505 bp wild-type region from *YGR290W* to right telomere, Repaired wild-type W region from *YGR254W* to *YGR246C* and *tN(GUU)G* gene was replaced back in yeast_chr07_9_02	Missing loxPsym sites: 409978-410011, 768480-768513, 953939-953972, 955439-955472.<br>Remaining TAG stop codons: 639475 A->G.<br>Wild-type PCRTags: 659213-659240 *YGR099W*_1_synF, 659567-659594 *YGR099W*_1_synR, 954240-954267 *YGR257C*_1_synF, 954705-954732 *YGR257C*_1_synR, 955686-955713 *YGR258C*_1_synF, 955950-955977 *YGR258C*_1_synR.<br>Point mutations that cause amino acid changes: 35647 G->A, 63955 T->C, 118823 G->A, 211372 A->T, 228617 G->A, 228865 G->A, 228978 A->G, 280790 A->T, 290392 C->T, 294107 G->A, 302039 C->T, 319501 G->A, 319502 G->A, 394086 T->C, 452503 A->G, 513232 A->G, 576521 T->A, 580950 A->C, 581229 A->G, 582191 T->C, 586977 A->T, 591572 T->C, 601536 T->C, 602470 G->A, 606435 A->AT, 606442 C->T, 638403 T->A, 638759 A->G, 640573 A->G, 640791 A->G, 641509 A->C, 642293 T->G, 728595 GA->G, 747229 C->T, 773455 C->G, 787845 G->T, 956885 A->G, 992161 A->G<br>deletion of *tN(GUU)G* at 694364 was replaced by the native sequence.
